# Bone Marrow-Specific Knock-In of a Non-Activatable Ikkα Kinase Mutant Influences Haematopoiesis but Not Atherosclerosis in *Apoe*-Deficient Mice

**DOI:** 10.1371/journal.pone.0087452

**Published:** 2014-02-03

**Authors:** Pathricia V. Tilstam, Marion J. Gijbels, Mohamed Habbeddine, Céline Cudejko, Yaw Asare, Wendy Theelen, Baixue Zhou, Yvonne Döring, Maik Drechsler, Lukas Pawig, Sakine Simsekyilmaz, Rory R. Koenen, Menno P. J. de Winther, Toby Lawrence, Jürgen Bernhagen, Alma Zernecke, Christian Weber, Heidi Noels

**Affiliations:** 1 Institute of Molecular Cardiovascular Research, RWTH Aachen University, Aachen, Germany; 2 Cardiovascular Research Institute Maastricht, Maastricht University, Maastricht, The Netherlands; 3 Department of Medical Biochemistry, Academic Medical Center, University of Amsterdam, Amsterdam, The Netherlands; 4 Centre d'Immunologie de Marseille-Luminy, Aix-Marseille Université, Marseille, France; 5 Institute of Biochemistry and Molecular Cell Biology, RWTH Aachen University, Aachen, Germany; 6 Institute for Cardiovascular Prevention, Ludwig-Maximilians-University Munich, Munich, Germany; 7 August-Lenz-Stiftung, Institute for Cardiovascular Research, Ludwig-Maximilians-University Munich, Munich, Germany; 8 Rudolf Virchow Center and Institute of Clinical Biochemistry and Pathobiochemistry, University of Würzburg, Würzburg, Germany; 9 Department of Vascular Surgery, Klinikum rechts der Isar Technical University Munich, Munich, Germany; 10 German Centre for Cardiovascular Research, partner site Munich Heart Alliance, Munich, Germany; National Jewish Health and University of Colorado School of Medicine, United States of America

## Abstract

**Background:**

The Ikkα kinase, a subunit of the NF-κB-activating IKK complex, has emerged as an important regulator of inflammatory gene expression. However, the role of Ikkα-mediated phosphorylation in haematopoiesis and atherogenesis remains unexplored. In this study, we investigated the effect of a bone marrow (BM)-specific activation-resistant *Ikkα* mutant knock-in on haematopoiesis and atherosclerosis in mice.

**Methods and Results:**

*Apolipoprotein E* (*Apoe*)-deficient mice were transplanted with BM carrying an activation-resistant *Ikkα* gene (*Ikkα^AA/AA^Apoe^−/−^*) or with *Ikkα^+/+^Apoe^−/−^* BM as control and were fed a high-cholesterol diet for 8 or 13 weeks. Interestingly, haematopoietic profiling by flow cytometry revealed a significant decrease in B-cells, regulatory T-cells and effector memory T-cells in *Ikkα^AA/AA^Apoe^−/−^* BM-chimeras, whereas the naive T-cell population was increased. Surprisingly, no differences were observed in the size, stage or cellular composition of atherosclerotic lesions in the aorta and aortic root of *Ikkα^AA/AA^Apoe^−/−^ vs Ikkα^+/+^Apoe^−/−^* BM-transplanted mice, as shown by histological and immunofluorescent stainings. Necrotic core sizes, apoptosis, and intracellular lipid deposits in aortic root lesions were unaltered. *In vitro*, BM-derived macrophages from *Ikkα^AA/AA^Apoe^−/−^ vs Ikkα^+/+^Apoe^−/−^* mice did not show significant differences in the uptake of oxidized low-density lipoproteins (oxLDL), and, with the exception of Il-12, the secretion of inflammatory proteins in conditions of Tnf-α or oxLDL stimulation was not significantly altered. Furthermore, serum levels of inflammatory proteins as measured with a cytokine bead array were comparable.

**Conclusion:**

Our data reveal an important and previously unrecognized role of haematopoietic Ikkα kinase activation in the homeostasis of B-cells and regulatory T-cells. However, transplantation of *Ikkα^AA^* mutant BM did not affect atherosclerosis in *Apoe^−/−^* mice. This suggests that the diverse functions of Ikkα in haematopoietic cells may counterbalance each other or may not be strong enough to influence atherogenesis, and reveals that targeting haematopoietic Ikkα kinase activity alone does not represent a therapeutic approach.

## Introduction

Cardiovascular diseases are the main cause of morbidity and mortality in western societies, with atherosclerosis being the underlying pathology triggering most of the cardio- and cerebrovascular incidents. Atherosclerosis is a chronic inflammatory disease of the vessel wall characterized by the activation of endothelial cells, the subendothelial accumulation of oxidized low-density lipoproteins (oxLDL) and the infiltration of inflammatory cells such as neutrophils, monocytes, dendritic cells (DCs) and lymphocytes [1], [2].

A key regulator of inflammation and atherogenesis is the transcription factor nuclear factor κB (NF-κB) [3]. The NF-κB family has 5 members: p65 (RelA), c-Rel, RelB, NF-κB1 (p105, processed to p50) and NF-κB2 (p100, processed to p52) [4]. Under resting conditions, canonical NF-κB dimers (mostly p50/p65) are predominantly found in the cytoplasm bound to the inhibitory of κB (IκB)-α protein. Inflammatory signals such as TNF-α and oxLDL activate the IκB kinase (IKK) complex, which consists of two catalytically active kinases (IKKα/IKK1 and IKKβ/IKK2) and one regulatory component (IKKγ/NEMO). The phosphorylation of IκB-α by IKKβ causes its ubiquitination and proteasomal degradation. This releases the NF-κB dimer, allowing a steady-state localization in the nucleus and the expression of many pro-inflammatory proteins [5]. These pro-inflammatory functions of canonical NF-κB activation have been linked to atherogenesis *in vivo*, showing reduced lesion formation in hyperlipidaemic *Apolipoprotein E* (*Apoe*)-deficient mice treated with the NF-κB inhibitor DHMEQ, which prevents the TNF-α-induced nuclear translocation of p65 [6]. Similarly, strongly reduced atherosclerotic plaque formation was observed in *Apoe^−/−^* mice with an endothelial cell-restricted inhibition of NF-κB activation through endothelial *Ikkγ*-deficiency or through an endothelial cell-specific expression of a dominant-negative *IκB-α* transgene [7]. On the other hand, NF-κB activity in leukocytes also has an important role in the resolution of inflammation through the transcription of anti-inflammatory cytokines such as interleukin (IL)-10 [8] and the suppression of pro-inflammatory IL1-β secretion [9]. Furthermore, an anti-inflammatory role was described for IKKβ by suppression of the classically activated (or M1) macrophage phenotype [10]. These anti-inflammatory properties of IKK/NF-κB signalling could explain the initially unexpected increase in atherosclerosis in hyperlipidaemic *Ldl receptor* (*Ldlr*)-deficient mice with a myeloid-specific deletion of *Ikkβ*, which was linked to a significant reduction of the anti-inflammatory cytokine IL-10 in *Ikkβ^−/−^* macrophages [11]. Thus, IKKβ/NF-κB signalling plays a complex role in inflammation and atherogenesis by driving both pro- and anti-inflammatory processes, and the outcome of NF-κB inhibition on atherosclerosis seems strongly dependent on the targeted cell type.

In contrast to IKKβ and IKKγ, the role of IKKα in atherogenesis has not been investigated. Although IKKα is not required for canonical IκB-α phosphorylation and subsequent NF-κB activation [12], [13], the IKKα kinase exerts multiple NF-κB-dependent and -independent functions that could potentially influence atherogenesis [4], [14]. First, IKKα homodimers mediate alternative NF-κB activation through phosphorylation of NF-κB p100, triggering p100 processing and the release and nuclear localization of RelB-p52 dimers [4], [15]. This pathway, induced by several members of the TNF-superfamily as BAFF and CD40 ligand, plays a central role in B-cell maturation and lymphoid organ formation and may thus implicate IKKα in atherogenesis through the recently appreciated role of B-cells in this pathology [16]. Secondly, nuclear IKKα modulates gene expression through phosphorylation of histone H3, mediating the expression of a subset of canonical NF-κB-dependent genes in TNF-α-stimulated mouse embryonic fibroblasts [17], [18]. On the other hand, IKKα has also been associated with repression or termination of gene transcription. For example, an anti-inflammatory role has been described for IKKα kinase in macrophages by inducing the phosphorylation, promoter removal and degradation of the canonical NF-κB isoforms p65 and c-Rel [19]. Furthermore, diverse pro-inflammatory stimuli trigger the IKKα-mediated phosphorylation of the transcriptional repressor PIAS1, which then negatively regulates the expression of a predominantly pro-inflammatory subset of p65- and STAT1-dependent genes [20]. In addition, IKKα-mediated phosphorylation of TAX1BP1 triggers the assembly of the A20 ubiquitin-editing complex, which is an important negative regulator of canonical NF-κB activation [21]. In conclusion, the IKKα kinase can positively or negatively regulate cell signalling and pro- and anti-inflammatory gene expression by phosphorylating diverse substrates, and could thus play a complex role in atherogenesis as well.

As haematopoietic cells are crucial players in the inflammatory reactions driving atherosclerosis, this study investigated the role of haematopoietic IKKα kinase activation in haematopoiesis and atherogenesis after transplantation of atherosclerosis-prone *Apoe^−/−^* mice with bone marrow carrying an activation-resistant *Ikkα^AA/AA^* mutant [22].

## Materials and Methods

### Nomenclature

The letter format of all gene and protein notations in this manuscript is in accordance with internationally agreed gene/protein nomenclature guidelines: all letters of human genes/proteins are in uppercase, whereas for mouse genes/proteins, only the first letter is in uppercase. Gene names are in italics.

### Mouse model, bone marrow (BM) transplantation and ethics statement

C57BL/6 *Ikkα^AA/AA^* mice, which are homozygous for an activation-resistant mutant of Ikkα through replacement of the serines 176/180 in the kinase activation loop with alanines [22], were crossed with atherosclerosis-prone C57BL/6 *Apoe^−/−^* to generate *Ikkα^AA/AA^ Apoe^−/−^* mice. BM cells (3×10^6^/mouse) from *Ikkα^AA/AA^Apoe^−/−^* mice or from *Ikkα^+/+^Apoe^−/−^* littermate controls were flushed from femur and tibia marrow cavities and were subsequently administered to female C57BL/6 *Apoe^−/−^* recipient mice by lateral tail vein injection one day after a lethal dose of whole-body irradiation (2×6.5 Gy). After four weeks of recovery, the mice were put on a high-fat diet containing 21% fat and 0.15% cholesterol (Altromin) for either 8 or 13 weeks, as indicated. All animal experiments were approved by local authorities (Landesamt für Natur, Umwelt und Verbraucherschutz Nordrhein-Westfalen, Germany; approval number 8.87-50.10.35.08.073) and complied with the German animal protection law. All surgery was performed under ketamine/xylazin anesthesia, and all efforts were made to minimize suffering.

### Determination of chimerism

The degree of chimerism in BM-transplanted mice was determined by quantifying the mutated *Ikkα^AA^* allele relative to the wild-type *Ikkα* allele in genomic DNA isolated from blood cells. Real-time quantitative PCR analysis was performed using the Maxima SYBR Green qPCR Mastermix (Fermentas) in a thermal cycler 7900HT (Applied Biosystems) using specific primer pairs (Sigma-Aldrich): 5′-CCTCTCAGTGGCTCACCTTT and 5′-CAATGTTCCCACAAAAGATGTACAGAGACT (for *Ikkα*); 5′-CCTCTCAGTGGCTCACCTTT and 5′-CAATGTTCCCACAAACGCTGTACAGAGCGC (for *Ikkα^AA^*); 5′-CAACGAGCGGTTCCGATG and 5′-GCCACAGGATTCCATACCCAA (for *β-Actin*). β-Actin was used as a reference gene. The method was validated by analyzing a standard curve using genomic DNA from *Ikkα^AA/AA^Apoe^−/−^* and *Ikkα^+/+^Apoe^−/−^* blood cells, mixed at different ratios.

### Lipid measurement in blood serum

Cholesterol and triglyceride levels in the blood serum were quantified using enzymatic assays (Cobas, Roche) according to the manufacturer's protocol. High-density (HDL), low-density (LDL) and very low-density (VLDL) cholesterol profiles were determined after HPLC-based fractionation of pooled serum samples using a Superose 10/300GL column followed by in-line cholesterol detection as described by Parini *et al.* [23].

### Haematopoietic profiling and serum cytokine quantification

Lymph nodes, spleen, thymus and BM were harvested and a single-cell suspension was prepared and filtered over a 70 µm cell strainer (Greiner). Splenocytes, BM cells and EDTA-buffered blood, obtained by retro-orbital puncture, were treated with erythrocyte lysis buffer (0.155 M NH_4_Cl, 10 mM NaHCO_3_) at room temperature. All cell suspensions were washed with HANKS Complete buffer containing 1× HBSS with 0.3 mM EDTA and 0.1% BSA, and stained with combinations of antibodies to Cd45, Cd11b, MhcII, Cd19, Cd3, Cd62L or Cd25 (BD Bioscience), to Cd11b, Cd115, Gr1, Cd3, Cd4, Cd8a, Cd25, Foxp3, Cd44, Cd11c or 440c (eBioscience), or to B220 (BioLegend). Intracellular labelling of Foxp3 was performed using the mouse regulatory T-cell staining kit (eBioscience) according to the manufacturer's protocol. Flow cytometric analysis was performed using a FACSCanto II and FACSDiva software (BD Biosciences) after appropriate fluorescence compensation, and leukocyte subsets were gated using FlowJo software (Treestar). B-cells were identified as Cd45^+^Cd19^+^ or B220^+^ as indicated; T-cells as Cd45^+^Cd3^+^; naive T-cells as Cd44^low^Cd62L^high^ T-cells; effector memory T-cells as Cd44^high^Cd62L^low^ T-cells; central memory T-cells as Cd44^high^Cd62L^high^ T-cells; regulatory T-cells (T_reg_ cells) as Cd4^+^Cd25^+^Foxp3^+^ T-cells; neutrophils as Cd45^+^Cd115^−^Gr1^high^; monocytes as Cd45^+^Cd115^+^; conventional DCs (cDCs) as Cd45^+^Cd11c^+^MhcII^+^; and plasmacytoid DCs (pDCs) as Cd45^+^Cd11c^+^Cd11b^−^440c^+^.

Cytokine levels in the blood serum were measured by flow cytometry using the BD Cytometric Bead Array Mouse Inflammation Kit (for Tnf-α, Mcp1, Il6, Ifn-γ, Il12 and Il10; BD-Pharmingen). In addition, Il10 levels were measured using a mouse Il10 Quantikine ELISA kit (R&D Systems).

### Atherosclerotic lesion analysis using histology and immunofluorescence staining

For atherosclerotic lesion analysis, the aortic root and thoraco-abdominal aorta were stained for lipid depositions with Oil-Red-O. In brief, the heart with aortic root was embedded in Tissue-Tek for cryo sectioning. Atherosclerotic lesions were quantified in 5 µm transverse sections and averages were calculated from 3–5 sections. The aorta was opened longitudinally, mounted on glass slides and en face-stained. Macrophages, smooth muscle cells (SMCs) and T-cells in the atherosclerotic lesions were visualized by immunofluorescent staining for Mac2 (Cedarlane), Sma (Dako) and Cd3 (AbD Serotec), respectively, followed by a FITC- or Cy3-conjugated secondary antibody staining (Jackson ImmunoResearch). Appropriate IgG antibodies were used as isotype controls. Nuclei were counterstained by 4′,6-diamidino-2-phenylindol (DAPI). Neutrophil presence was examined by Naphthol-AS-D-chloroacetate Esterase (ASDCL) staining. Apoptotic nuclei were detected by terminal deoxynucleotidyl nick-end labelling (TUNEL-kit, Roche). Intracellular lipid deposits in aortic root lesions were stained using Nile Red (N-3013, Sigma). All images were recorded with a Leica DMLB fluorescence microscope and CCD camera. The quantification of lesion size and composition was performed using Diskus analysis software (Hilgers), whereas the Nile Red stainings were analyzed with help of Image J software. All analyses were performed without prior knowledge of the genotype.

To explore potential qualitative effects on atherosclerosis, the aortic root lesions were classified according to phenotype, as previously described [11]. Three categories were distinguished: (1) early lesions, containing only foam cells, (2) intermediate-type lesions, presenting foam cells, some necrosis and a fibrotic cap, (3) advanced lesions, showing extended fibrosis and necrosis and infiltration of the plaque into the media.

### Preparation and labelling of oxLDL

OxLDL was prepared by oxidation of LDL (Calbiochem) with a 50 nM copper sulphate solution for 4 hours at 37°C for mildly oxidized LDL and overnight for heavily oxidized LDL, followed by purification over a PD 10 column (GE Healthcare). For lipid uptake experiments heavily oxidized LDL was labelled with 1,10-dioctadecyl-3,3,3030-tetramethylindocyanide percholorate (Dil). After incubation of 0,5 mg/ml oxLDL with 20 µL of Dil (stock 3 mg/ml in DMSO) overnight at 37°C, the Dil-labelled oxLDL was purified over a PD 10 column and stored at 4°C for a maximum of 2 weeks.

### BM-derived macrophages, nuclear extracts and NF-κB p65 DNA-binding ELISA

BM-derived macrophages were generated as previously described [24]. Briefly, BM from the femurs and tibiae was flushed with ice-cold PBS using a 27-G needle, resuspended in PBS by repeated vigorous pipetting and filtered using a 70 µM cell strainer (BD Biosciences). The filtered solution was centrifuged, the resulting pellet resuspended in culture medium and the cells plated on 15 cm untreated culture dishes (Greiner). BM-derived macrophages from cryopreserved BM-cells were generated as previously described [25]. Culturing and stimulation of BM-derived macrophages were performed in RPMI 1640 (+L-Glutamin) containing 10 mM Hepes, 10% FCS, 15% L929-cell-conditioned medium and 100 U/ml gentamycin. After 7 days of culturing, differentiated macrophages were used for stimulation experiments and transferred onto untreated 6-well dishes (Greiner). The cells were left for 24 hours to adhere und were then stimulated with 100 ng/ml lipopolysaccharide (LPS) (Sigma-Aldrich), 10 ng/ml mouse Tnf-α (Peprotech) or 50 µg/ml mildly oxidized LDL, as indicated.

Nuclear extracts were isolated as described [11]. Briefly, BM-derived macrophages were washed once with PBS and scraped of the culture dish using PBS with 5 mM EDTA. The cells were centrifuged and the cell pellet was resuspended in 100 µl buffer A (10 mM Hepes pH 7.8, 1.5 mM MgCl_2_, 0.5 mM DTT and 1× Complete EDTA-free protease inhibitor cocktail (Roche)). After 2 min incubation on ice, 100 µl of buffer A supplemented with 1.28% NP40 was added to the cell suspension and incubated on ice for 10 min. The cells were vortexed for 10 sec and centrifuged for 10 min at 2000 rpm. The supernatant was removed, the pellet dissolved in 50 µl buffer B (20 mM Hepes pH 7.8, 420 mM NaCl; 1,2 mM MgCl_2_, 0.2 mM EDTA, 25% glycerol, 0.5 mM DTT and 1× Complete EDTA-free protease inhibitor cocktail (Roche)) and incubated for 30 min on ice. Every 5 min, the sample was vortexed thoroughly. Then the solution was centrifuged for 15 min at 4000 rpm and the supernatant, being the nuclear lysate, was snap-frozen at −80°C. Protein concentration was determined using the Quick Start Bradford Protein Assay (Biorad).

NF-κB p65 DNA-binding activity in equal amounts of nuclear extracts was quantified using an oligonucleotide-based ELISA (TransAM NF-κB p65 ELISA, Active Motif) according to the supplier's instructions. Absorbance values were corrected for background by incubation with lysis buffer only.

### *In vitro* macrophage foam cell formation and cytokine secretion

To quantify the uptake of DiI-labelled heavily oxidized LDL by BM-derived macrophages, macrophages were plated on 24-well plates and incubated overnight at 37°C. The next day, non-adherent cells were rinsed off with PBS and medium containing 1 µg/ml or 10 µg/ml Dil-oxLDL was added. To analyze whether oxLDL uptake occurred in an actin-dependent way, controls were pre-incubated for 1 h with 10 mM cytochalasin D, followed by a stimulation with 10 µg/ml Dil-oxLDL and 10 mM cytochalasin D. The cells were stimulated for 3 or 24 hours as indicated, washed with PBS and stained with F4/80 (clone BM8, eBioscience). Flow cytometric analysis was performed using a FACSCanto II and the data were analyzed using FlowJo software (Treestar). Data were calculated by subtracting the cell autofluorescence (cells without diI-oxLDL incubation) from the fluorescence of the diI-oxLDL-treated samples and were expressed as geometric mean fluorescence intensity (gMFI).

To measure cytokine and chemokine secretion from BM-derived macrophages, cells were plated in 6-well plates, left for 24 h to adhere und were then stimulated with 10 ng/ml mouse Tnf-α (Peprotech) or 50 µg/ml heavily oxidized LDL. As control unstimulated cells were included. After 24 h the medium was harvested from the cells, and cytokine and chemokine levels were measured by flow cytometry using the BD Cytometric Bead Array Mouse Inflammation Kit (BD-Pharmingen). In addition, Mcp1 levels were measured using a mouse Mcp1 ELISA (DY479, R&D Systems).

### Statistical analysis

All statistical analyses were performed using GraphPad Prism (GraphPad Software Inc.). Data are represented as means ± SEM and were analyzed by 2-tailed Student's t-test or 2-way ANOVA with Bonferroni post-test, as appropriate. P<0.05 was considered statistically significant.

## Results

### Bone marrow-specific loss of Ikkα kinase activation affects B- and T-lymphocyte populations

To study the role of IKKα kinase activation in haematopoiesis in the context of atherosclerosis, BM was isolated from *Ikkα^+/+^Apoe^−/−^* or *Ikkα^AA/AA^Apoe^−/−^* mice, the latter carrying an non-activatable mutant of Ikkα through replacement of serines 176/180 in its activation loop with alanine residues [22]. After transplantation of this BM into lethally irradiated *Apoe^−/−^* mice and a recovery period of four weeks, the mice received a high-cholesterol diet for 13 weeks to accelerate atherosclerosis. Quantitative real-time PCR for the *Ikkα^AA/AA^ vs Ikkα^+/+^* allele in white blood cells of the transplanted recipient mice indicated that on average 96.1% (±1.2) of leukocytes carried the mutant *Ikkα^AA/AA^* allele, confirming a successful engraftment.

**Figure 1:**
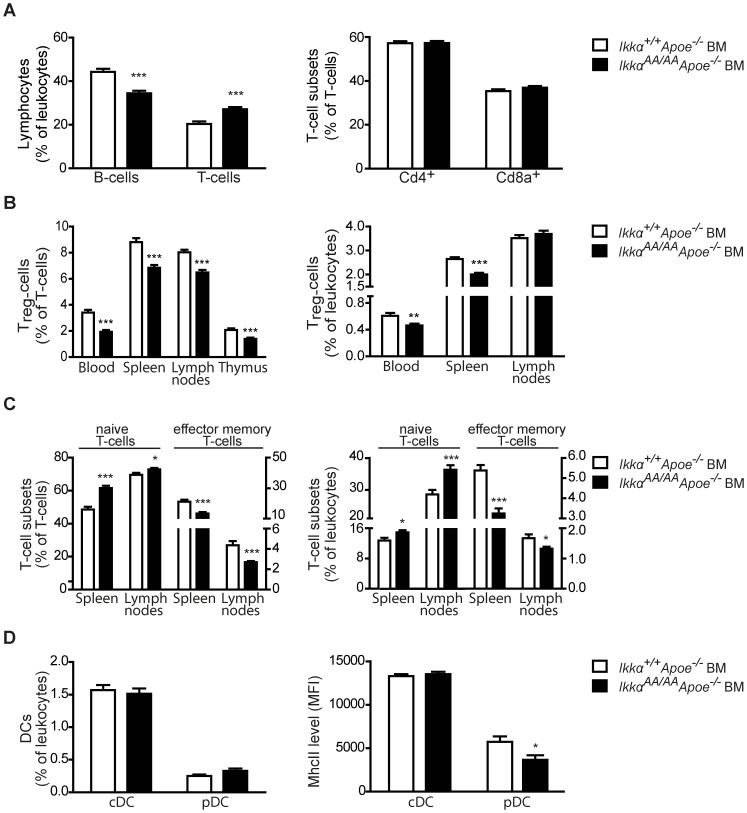
*Ikkα^AA/AA^Apoe^−/−^* BM-chimeras have less B-cells, T_reg_ and effector memory T-cells, and more naive T-cells. Shown is flow cytometric analysis of peripheral blood, thymus and secondary lymphoid organs of *Apoe^−/−^* mice transplanted with *Ikkα^AA/AA^Apoe^−/−^* or *Ikkα^+/+^Apoe^−/−^* BM and receiving a high-cholesterol diet for 13 weeks. (**A**) Cd19^+^ B-cell and Cd3^+^ T-cell populations as percentage of Cd45^+^ leukocytes, and Cd4^+^ and Cd8a^+^ T-cell subsets as percentage of Cd3^+^ T-cells in peripheral blood. (**B**) Cd3^+^Cd4^+^Cd25^+^Foxp3^+^ regulatory T-cell (T_reg_) levels as percentage of Cd3^+^ T-cells and Cd45^+^ leukocytes. (**C**) Cd3^+^Cd44^low^Cd62L^high^ naive T-cells and Cd3^+^Cd44^high^Cd62L^low^ effector memory T-cells as percentage of Cd3^+^ T-cells and Cd45^+^ leukocytes. Left Y-axes belong to naive T-cells, right Y-axes to effector memory T-cells. (**D**) Cd11c^+^MhcII^+^ conventional dendritic cells (cDCs) and Cd11c^+^Cd11b^−^440c^+^ plasmacytoid DCs (pDCs) as percentage of Cd45^+^ leukocytes *(left)*. Surface expression of MhcII on splenic cDCs and pDCs *(right)*. (**A–D**) All graphs represent the mean ± SEM (n = 18–19); 2-tailed t-test, *P<0.05, **P<0.01, ***P<0.001.

Interestingly, although total leukocyte counts were unaffected, *Ikkα^AA/AA^Apoe^−/−^* BM chimeras showed a significantly reduced Cd19^+^ B-cell population in peripheral blood (Figure 1A, Table 1). A similar decrease in B-cell number was seen in the BM and lymph nodes of the *Ikkα^AA/AA^Apoe^−/−^* BM chimeras, whereas no difference was observed in the spleen B-cell population (Figure S1).

**Table 1:** Leukocyte numbers and frequencies of leukocyte subsets in *Apoe^−/−^* mice transplanted with *Ikkα^AA/AA^Apoe^−/−^* or *Ikkα^+/+^Apoe^−/−^* BM.

	Ikkα^+/+^Apoe^−/−^ BM	Ikkα^AA/AA^Apoe^−/−^ BM
Leukocytes (×10^3^/µl blood)	7.9±0.6	7.1±0.3
Monocytes (% of leukocytes)	7.9±0.5	8.7±0.6
Gr1^high^ monocytes (% of monocytes)	66.0±2.0	68.1±2.7
Gr1^high^ monocytes (% of leukocytes)	5.2±0.3	5.7±0.3
Gr1^low^ monocytes (% of monocytes)	29.8±2.1	26.9±2.4
Gr1^low^ monocytes (% of leukocytes)	2.4±0.3	2.5±0.3
Neutrophils (% of leukocytes)	19.0±1.3	20.4±1.2

Peripheral blood leukocyte counts were determined after 8 weeks of high-fat diet (n = 8). Leukocyte subset frequencies were quantified by flow cytometry after 13 weeks of high-fat diet (n = 18–19).

In addition, also Cd3^+^Cd4^+^Cd25^+^Foxp3^+^ regulatory T-cells (T_reg_), which are associated with atheroprotection [26], were found to be significantly reduced in the Cd3^+^ T-cell population of the peripheral blood, thymus and secondary lymphoid organs of the *Ikkα^AA/AA^Apoe^−/−^* -transplanted mice (Figure 1B). Also among Cd45^+^ leukocytes, T_reg_ frequency was markedly decreased in blood and spleens of the *Ikkα^AA/AA^Apoe^−/−^* BM chimeras (Figure 1B). However, this was not observed in the lymph nodes (Figure 1B), which showed a significant overall increase of Cd3^+^ T-cells in the leukocyte population (Figure S1B).

No changes were observed in the relative proportions of Cd4^+^ and Cd8a^+^ T-cell subsets in blood or lymphoid organs (Figure 1A, Figure S1). However, spleen and lymph nodes of *Ikkα^AA/AA^Apoe^−/−^*-transplanted mice displayed a significant increase in the Cd3^+^Cd44^low^Cd62L^high^ naive T-cell population, whereas Cd3^+^Cd44^high^Cd62L^low^ effector memory T-cells, which mediate effector functions in inflamed tissue [27], were significantly reduced (Figure 1C, Figure S2). Also, the Cd3^+^Cd44^high^Cd62L^high^ central memory T-cell population, associated with the successive production of effector T-cells [27], was significantly decreased among splenic T-lymphocytes and total leukocytes (Figure S3).

Furthermore, relative frequencies of Cd115^−^Gr1^+^ neutrophils, Cd115^+^ monocytes and both Gr1^high^ and Gr1^low^ monocyte subsets in the peripheral blood were unchanged (Table 1). Also, splenocytes showed comparable frequencies of Cd11c^+^MhcII^+^ conventional dendritic cells (cDCs) and Cd11c^+^Cd11b^−^440c^+^ plasmacytoid DCs (pDCs) (Figure 1D). Nonetheless, expression of the activation marker MhcII was significantly reduced on splenic pDCs of *Ikkα^AA/AA^Apoe^−/−^* BM chimeras (Figure 1D).

A similar effect on B- and T-cell populations was observed in a non-atherosclerotic context. C57BL/6 mice transplanted with *Ikkα^AA/AA^* BM displayed a significantly reduced B-cell population in lymph nodes compared to controls, whereas both Cd4^+^ and Cd8a^+^ T-cell subsets were increased (Figure S4A,B). Similarly as observed before, T_reg_ lymphocytes were markedly decreased (Figure S5). Altogether, these findings indicate an important role for Ikkα kinase activity in haematopoiesis, with reduced B-cells and T_reg_ lymphocytes upon haematopoietic expression of an Ikkα^AA/AA^ mutant.

### Atherosclerotic lesions are unaltered in *Ikkα^AA/AA^Apoe^−/−^* bone marrow chimeras

**Figure 2:**
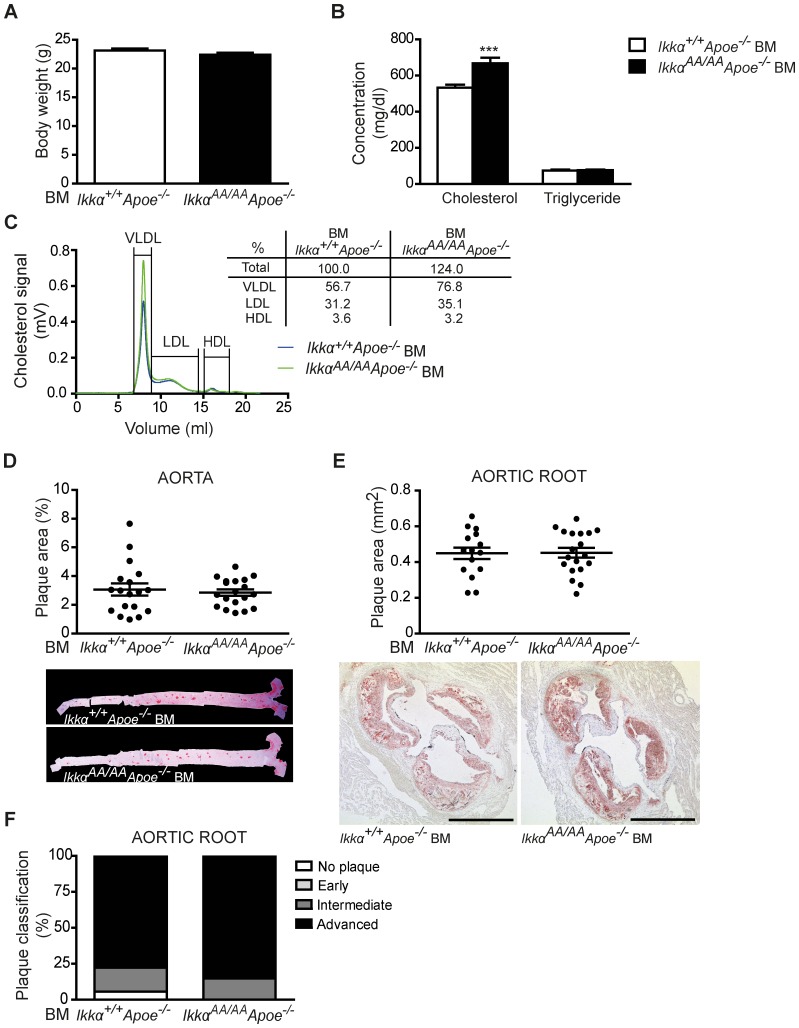
Knock-in of *Ikkα^AA/AA^* in haematopoietic cells does not influence advanced atherosclerosis in *Apoe^−/−^* mice. *Apoe^−/−^* mice were transplanted with *Ikkα^AA/AA^Apoe^−/−^* or *Ikkα^+/+^Apoe^−/−^* BM and received a high-cholesterol diet for 13 weeks before analysis. (**A,B**) Body weight (A) and lipid analysis in blood serum (B) (n = 18). (**C**) Cholesterol levels after HPLC-based size fractionation of pooled blood serum samples. (**D,E**) Atherosclerotic lesion sizes in the aorta (D) and aortic root (E) (n = 16–19). Representative pictures of Oil-Red-O^+^ lipid depositions in aorta and aortic root are shown. Scale bar = 500 µm. (**F**) Classification of aortic root lesions according to their severity. Shown is the plaque distribution as percentage of the total number of lesions examined (n = 18–27). (A,B,D,E) Graphs represent the mean ± SEM; 2-tailed t-test, ***P<0.001.

**Figure 3:**
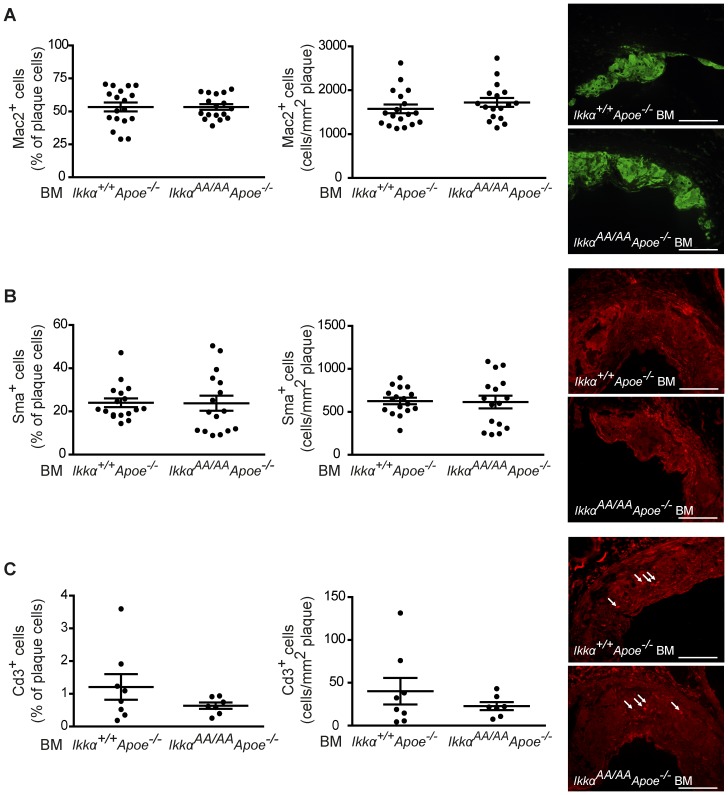
Knock-in of *Ikkα^AA/AA^* in haematopoietic cells does not influence the cellular composition of atherosclerotic lesions. Immunofluorescent stainings of aortic roots from *Apoe^−/−^* mice reconstituted with *Ikkα^AA/AA^Apoe* ^−/−^ or *Ikkα^+/+^Apoe* ^−/−^ BM and receiving a high-fat diet for 13 weeks. (**A–C**) Quantification of Mac2^+^ macrophages (A, n = 17–18), Sma^+^ SMCs (B, n = 16–17) and Cd3^+^ T-cells (C, n = 7–8) as percentage of all plaque cells *(left)* and relative to the plaque area *(right)*. Graphs represent the mean ± SEM. Representative pictures are shown. Scale bar = 100 µm.

**Figure 4:**
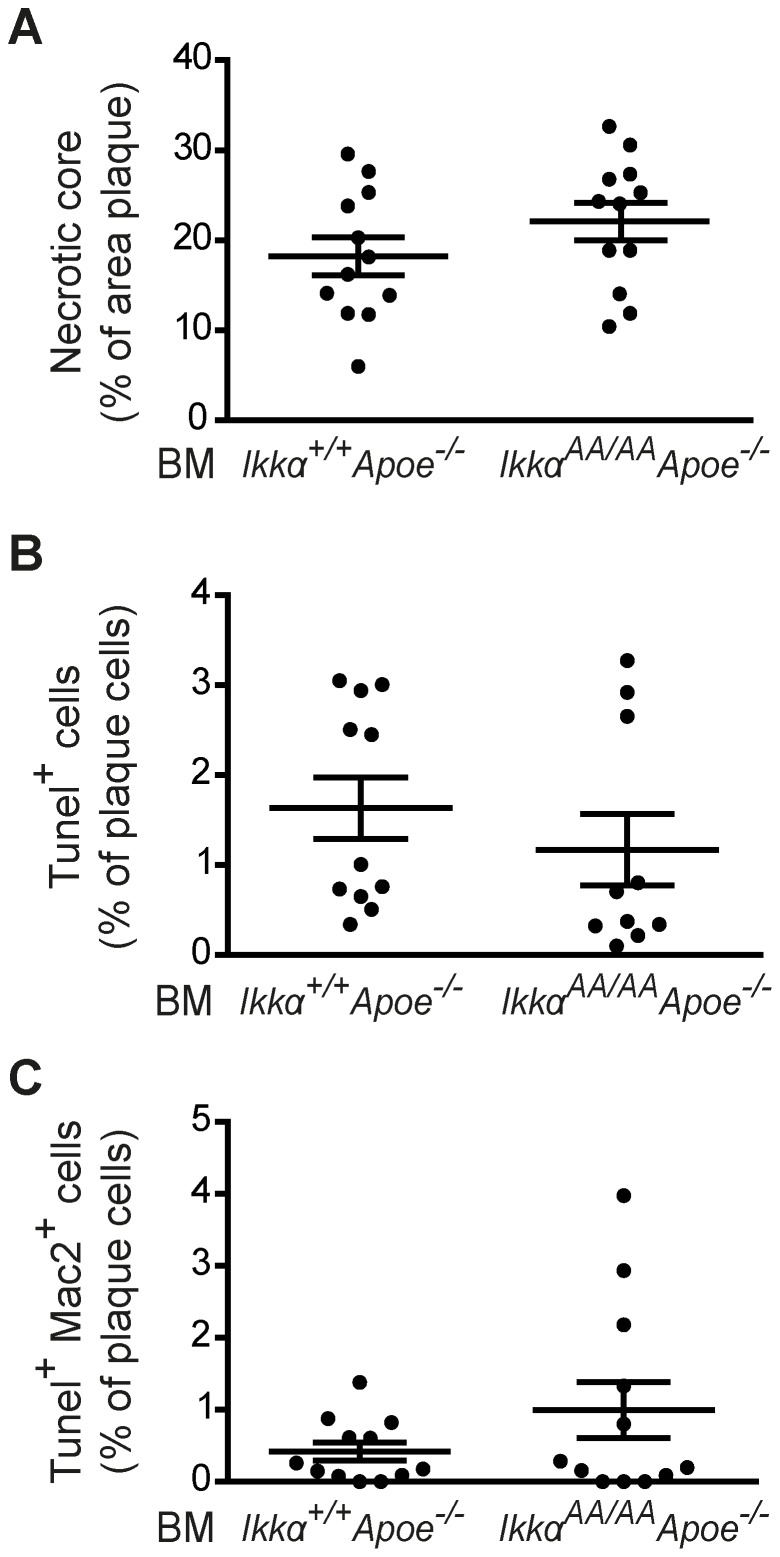
Knock-in of *Ikkα^AA/AA^* in haematopoietic cells does not influence apoptosis in atherosclerotic lesions. Analysis of aortic root lesions of *Apoe^−/−^* mice transplanted with *Ikkα^AA/AA^Apoe^−/−^* or *Ikkα^+/+^Apoe^−/−^* BM and receiving a high-fat diet for 13 weeks. (**A**) Quantification of necrotic cores as percentage of plaque area. (B–C) Quantification of apoptotic cells (Tunel^+^, B) and apoptotic macrophages (Tunel^+^Mac2^+^, C) as percentage of all plaque cells. Graphs represent the mean ± SEM (n = 10–12).

After 13 weeks of high-cholesterol diet, *Ikkα^AA/AA^Apoe^−/−^* and *Ikkα^+/+^Apoe^−/−^* BM chimeras were sacrificed and lipid levels in serum and the extent of atherosclerosis in the aorta and aortic root were analyzed. Whereas body weight and serum triglyceride values were similar, cholesterol levels were significantly increased in *Ikkα^AA/AA^Apoe^−/−^ vs Ikkα^+/+^Apoe^−/−^* BM-transplanted *Apoe^−/−^* mice (Figure 2A,B). This could be attributed to an increase in VLDL and LDL lipoprotein fractions, as shown by a cholesterol analysis after HPLC-based lipoprotein size separation of pooled serum samples (Figure 2C). Despite these differences in cholesterol levels, atherosclerotic lesion sizes in the aorta and aortic root were comparable in *Ikkα^AA/AA^Apoe^−/−^* and *Ikkα^+/+^Apoe^−/−^* BM chimeras (Figure 2D,E). To explore potential qualitative effects on atherosclerosis, the aortic root lesions were phenotypically classified into early, intermediate-type and advanced lesions, as previously described [11]. Again, no differences were found between both groups (Figure 2F). Also, the cellular composition of aortic root lesions was comparable, presenting an equal content of macrophages (Mac2^+^) and SMCs (Sma^+^) as shown by immunofluorescent stainings (Figure 3A,B). Similarly, no significant differences were observed in lesional Cd3^+^ T-lymphocyte content (Figure 3C). No neutrophils could be detected in any of the aortic root lesions. Finally, necrotic core sizes were comparable (Figure 4A), and no significant differences were found in the content of TUNEL^+^ apoptotic cells and apoptotic macrophages in aortic root lesions of *Ikkα^AA/AA^Apoe^−/−^* and *Ikkα^+/+^Apoe^−/−^* BM chimeras (Figure 4B,C).

**Figure 5:**
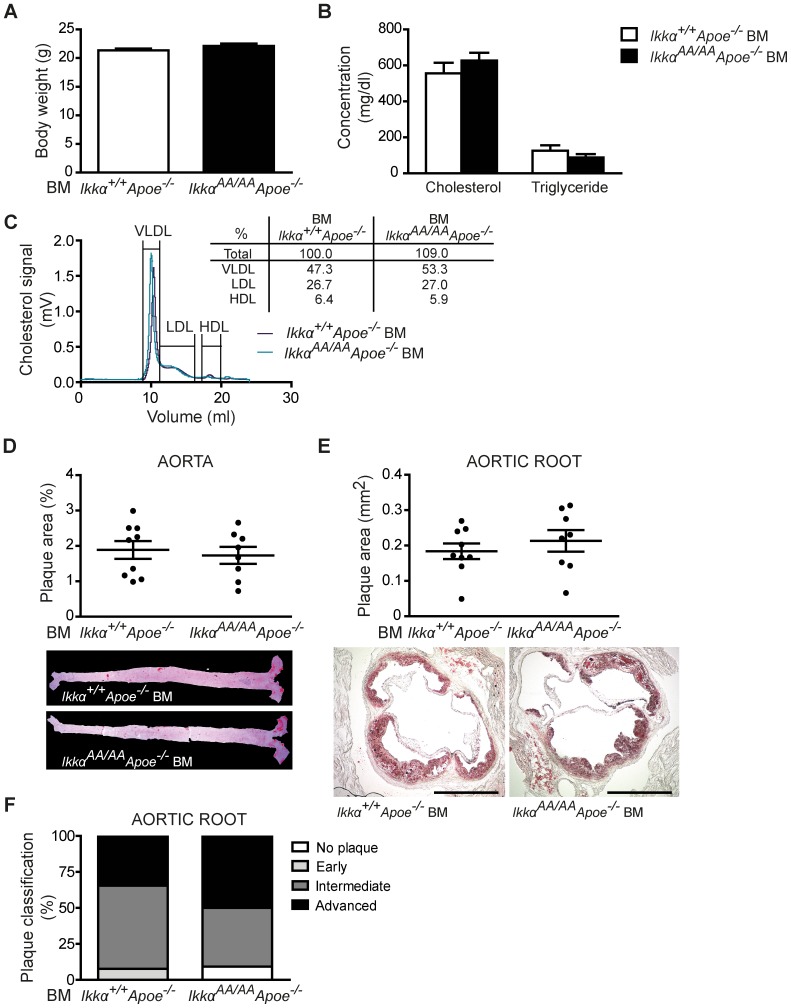
*Ikkα^AA/AA^* knock-in in haematopoietic cells does not influence earlier stages of atherogenesis in *Apoe^−/−^* mice. *Apoe^−/−^* mice were transplanted with *Ikkα^AA/AA^Apoe^−/−^* or *Ikkα^+/+^Apoe^−/−^* BM and received a high-cholesterol diet for 8 weeks before analysis. (**A,B**) Body weight (A) and lipid analysis in blood serum (B) (n = 7–9). (**C**) Cholesterol levels after HPLC-based size fractionation of pooled blood serum samples. (**D,E**) Atherosclerotic lesion sizes in the aorta (D) and aortic root (E). Representative pictures of Oil-Red-O^+^ lipid depositions in aorta and aortic root are shown. Scale bar = 500 µm. (**F**) Classification of aortic root lesions according to their severity. Shown is the plaque distribution as percentage of the total number of lesions examined (n = 22–26). (A,B,D,E) Graphs represent the mean ± SEM.

To investigate potential effects on earlier stages of atherosclerosis, a similar study was performed after a high-cholesterol diet fed for only 8 weeks. Successful engraftment of *Ikkα^AA/AA^Apoe^−/−^* BM into lethally irradiated *Apoe^−/−^* mice was again confirmed by quantitative real-time PCR, showing the mutant *Ikkα^AA/AA^* allele to be present in 94.3% (±2.7) of blood leukocytes. After this shorter period of high-fat diet, no differences were obtained in body weight or lipid levels in the blood serum, and also HPLC-based lipoprotein fractionation of pooled serum samples showed comparable HDL-, LDL- and VLDL-associated cholesterol peaks (Figure 5A–C). Similarly as for the 13 week-high-fat diet, *Ikkα^AA/AA^Apoe^−/−^* and *Ikkα^+/+^Apoe^−/−^* BM chimeras presented comparable atherosclerotic lesion sizes in the aorta and aortic root (Figure 5D,E). Lesions were reduced in size with 39% and 56% in aorta and aortic root, respectively, compared to the longer diet course, as can be expected. Also, classification of the plaques into early, intermediate or advanced stages revealed comparable lesion phenotypes in *Ikkα^AA/AA^Apoe^−/−^* and *Ikkα^+/+^Apoe^−/−^* BM-transplanted *Apoe^−/−^* mice, although the lesions were less advanced compared to the 13 week-diet study (Figure 5F).

In summary, these data indicated that the transplantation of *Ikkα^AA/AA^Apoe^−/−^* -mutant BM does not affect the size, phenotype or cellular content of atherosclerotic lesions in atherosclerosis-prone *Apoe^−/−^* mice.

### Haematopoietic knock-in of the *Ikkα^AA/AA^* mutant does not affect systemic inflammatory gene expression in *ApoE*-deficient mice

**Figure 6:**
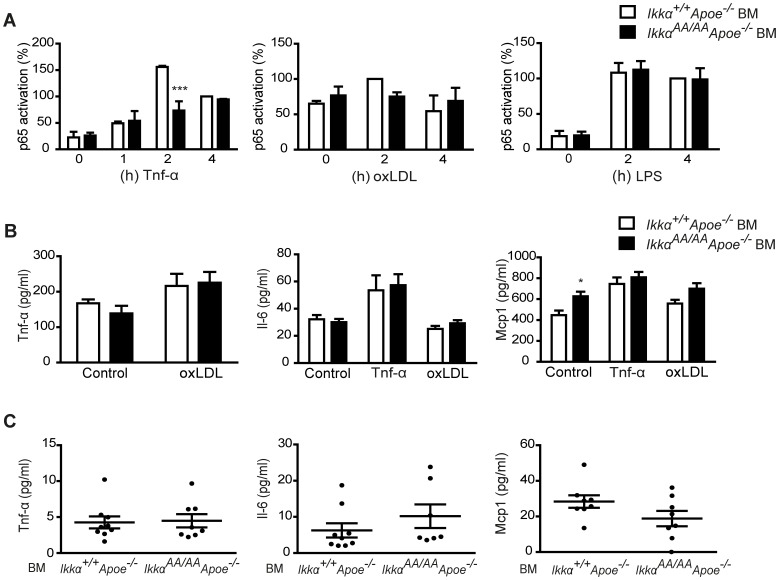
*Ikkα^AA/AA^* knock-in does not enhance or prolong NF-κB p65 activity, or majorly influence cytokine expression in *Apoe^−/−^* macrophages *in vitro*. (A) BM-derived macrophages from *Ikkα^AA/AA^Apoe^−/−^* and *Ikkα^+/+^Apoe^−/−^* mice were stimulated *in vitro* with 10 ng/ml Tnf-α, 50 µg/ml of mildly oxidized LDL or 100 ng/ml LPS for the indicated time. Activation of p65 was quantified in nuclear extracts using a TransAm p65 assay. Graphs represent the mean ± SEM (n = 2); 2-way ANOVA with Bonferroni post-test, ***P<0.001. (B) BM-derived macrophages from *Ikkα^AA/AA^Apoe^−/−^* and *Ikkα^+/+^Apoe^−/−^* mice were stimulated *in vitro* for 24 h with 10 ng/ml Tnf-α or 50 µg/ml heavily oxidized LDL. Cytokine concentrations in the supernatants are displayed for Tnf-α, Il-6 and Mcp1. Graphs represent mean ± SEM (n = 9 from 3 independent experiments); 2-way ANOVA with Bonferroni post-test, ***P<0.001. (C) Concentrations of Tnf-α, Mcp1 and Il-6 in serum of *Apoe^−/−^* mice transplanted with *Ikkα^AA/AA^Apoe^−/−^* or *Ikkα^+/+^Apoe^−/−^* BM and receiving a high-fat diet for 8 weeks. Graphs represent the mean ± SEM (n = 7–9).

The canonical NF-κB pathway has been shown to play an important role in controlling atheroprogression, at least partly by balancing anti- and pro-inflammatory processes in macrophages [11]. Canonical NF-κB activation is dependent on IKKβ and IKKγ, but does not require IKKα kinase activity for IKK-mediated IκB-α phosphorylation and degradation with subsequent NF-κB activation [12], [13]. On the other hand, IKKα has been shown to terminate LPS-induced NF-κB activity in macrophages by promoting the phosphorylation and degradation of the NF-κB isoform p65, thereby abrogating p65-mediated gene transcription [19]. Therefore, we aimed to examine the direct effect of the *Ikkα^AA/AA^* knock-in mutation on canonical NF-κB activity in the context of atherosclerosis by studying the DNA binding capacity of p65 in nuclear extracts of *Ikkα^AA/AA^Apoe^−/−^ vs Ikkα^+/+^Apoe^−/−^* BM-derived macrophages *in vitro*. Unexpectedly, the *Ikkα^AA/AA^* knock-in mutation did not enhance or prolong p65 activity in *Apoe*-deficient macrophages upon stimulation with Tnf-α or oxidized LDL (oxLDL), i.e. mimicking inflammatory or atherogenic challenges, and even slowed down Tnf-α-induced p65 activation. No differences were observed in LPS-induced p65 activity in *Ikkα^AA/AA^Apoe^−/−^ vs Ikkα^+/+^Apoe^−/−^* BM-derived macrophages (Figure 6A).

Furthermore, the concentrations of the inflammatory proteins Tnf-α, Il-6 and Mcp1 in supernatants of Tnf-α- or oxLDL-stimulated *Ikkα^AA/AA^Apoe^−/−^* and *Ikkα^+/+^Apoe^−/−^* BM-derived macrophages were not significantly different, although *Ikkα^AA/AA^* knock-in significantly enhanced the basal secretion of Mcp1, in contrast to Tnf-α or Il-6 (Figure 6B). Neither Tnf-α nor oxLDL were able to induce secretion of Il-10 or Il-12p70 *in vitro*, although Il-12p70 secretion was found to be significantly higher in oxLDL-stimulated macrophages upon *Ikkα^AA/AA^* knock-in (Figure S6). In addition, quantification of Tnf-α, Il-6 and Mcp1 in serum of *Ikkα^AA/AA^Apoe^−/−^ vs Ikkα^+/+^Apoe^−/−^* BM chimeras after 8 weeks of high-fat diet did not reveal significant differences (Figure 6C), whereas the cytokines Ifn-γ, Il-12 and Il-10 remained below the detection limit in both groups.

**Figure 7:**
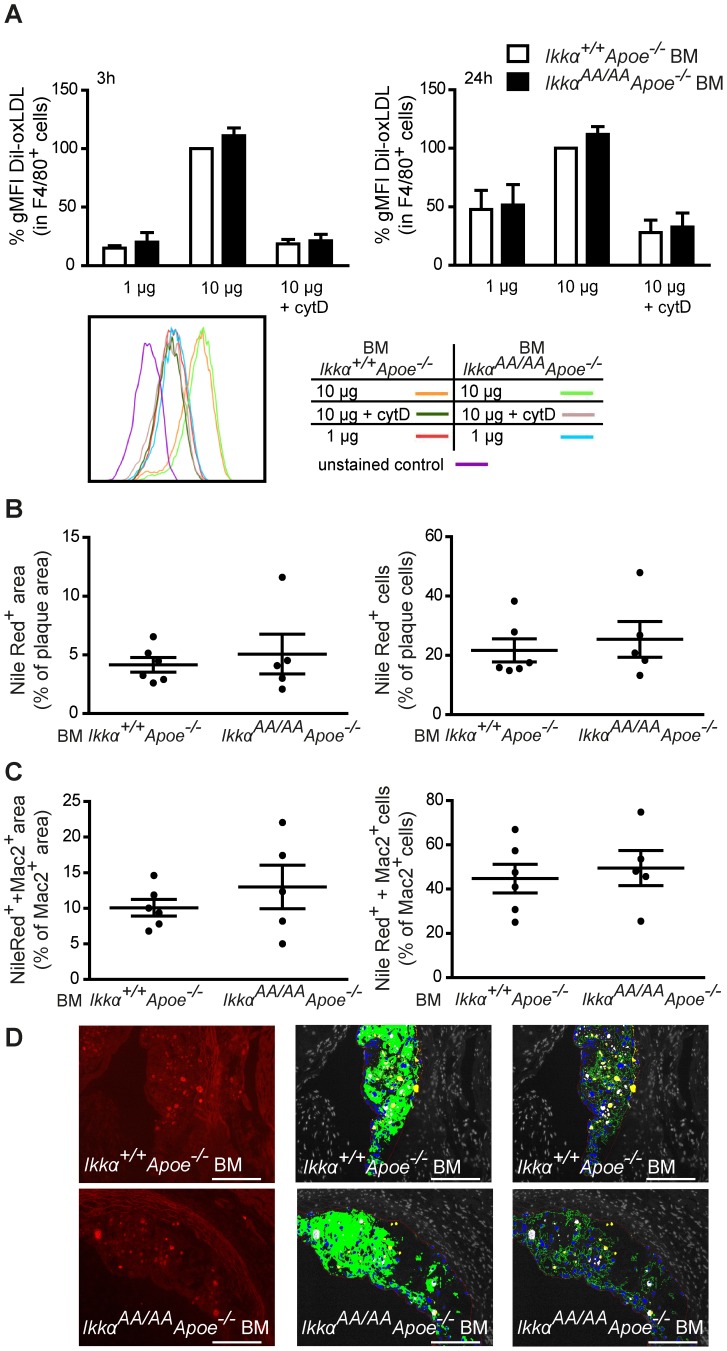
*Ikkα^AA/AA^* knock-in does not influence macrophage lipid uptake. (**A**) Flow cytometric analysis of Dil-oxLDL uptake by BM-derived macrophages. Cells were incubated without or with Dil-oxLDL (1 µg/ml or 10 µg/ml) for 3 h or 24 h, as indicated. To test for actin-dependent uptake, additional controls were simultaneously treated with cytochalasin D (cytD), as indicated. Representative flow cytometric histograms are shown. Graphs represent the mean ± SEM (n = 3); 2-way ANOVA with Bonferroni post-test. (**B–D**) Intracellular lipid depositions in aortic root lesions were stained with Nile Red and co-stained with Mac2 in order to quantify lipid-laden macrophages. (B) Nile Red staining was quantified relative to the plaque area (left graph), and Nile Red^+^ cells were quantified as percentage of total plaque cells (right graph). (C) Lipid uptake by macrophages was quantified as Nile Red^+^Mac2^+^ area as % of Mac2^+^ area (left graph), and as Nile Red^+^Mac2^+^ cells as % of Mac2^+^-cells. (D) Shown are representative pictures from Nile Red staining (red fluorescence; left image). The middle and right image demonstrate Image J analyses. Displayed are the plaque area (thin red line), macrophage area (green), plaque cell nuclei (blue), Nile Red^+^ area (yellow) and Mac2^+^ Nile Red^+^ area (white, lipid deposits in macrophages). Graphs represent the mean ± SEM (n = 5–6).

Given the importance of lipid uptake by macrophages in atherosclerotic lesions, we examined a possible effect of *Ikkα^AA/AA^* knock-in on macrophage foam cell formation *in vitro*. With 2 different incubation times and oxLDL doses, we did not observe a significant difference in oxLDL uptake by *Ikkα^AA/AA^Apoe^−/−^ vs Ikkα^+/+^Apoe^−/−^* BM-derived macrophages (Figure 7A). Cytochalasin D severely reduced the oxLDL-associated fluorescence signal, indicating that oxLDL was actively taken up by the cells in an actin-dependent way and not merely binding the cell surface (Figure 7A). Next, we quantified lipid deposits in aortic root lesions of *Ikkα^AA/AA^Apoe^−/−^* and *Ikkα^+/+^Apoe^−/−^* BM chimeras after 13 weeks of high-cholesterol diet using Nile Red staining, but no difference were observed (Figure 7B,D). Also, quantification of Nile Red-positive macrophages after co-staining for Mac2 together with Nile Red dye revealed comparable intracellular lipid deposits in lesional macrophages and an equal amount of lipid-laden macrophages (Figure 7C,D).

In conclusion, these data show that knock-in of *Ikkα^AA/AA^* in an *Apoe^−/−^* background does not enhance NF-κB activity or majorly influence the secretion of important inflammatory proteins from Tnf-α- or oxLDL-stimulated macrophages, nor does it significantly affect macrophage foam cell formation.

## Discussion

Atherosclerosis is characterized by a chronic inflammation of the vessel wall and all leukocyte subsets, including B- and T-lymphocytes, monocytes and monocyte-derived macrophages, neutrophils and DCs, contribute in their own specific ways to the pathogenesis of this widespread disease [1], [2]. Therefore, we investigated the effect of a BM-specific non-activatable *Ikkα^AA^* knock-in on haematopoiesis in conditions of atherosclerosis and identified a significant reduction in the B-cell population in the blood and lymph nodes of hyperlipidaemic *Ikkα^AA/AA^Apoe^−/−^* BM chimeras in comparison with *Ikkα^+/+^Apoe^−/−^* BM-transplanted *Apoe^−/−^* controls (Figure 1A, Figure S1). Comparable results were obtained in a non-atherosclerotic context, with a significantly reduced B-cell population in lymph nodes of C57BL/6 mice transplanted with *Ikkα^AA/AA^ vs Ikkα^+/+^* BM (Figure S4), and are consistent with earlier observations of a reduced mature B-cell population in secondary lymphoid organs of *Ikkα^AA/AA^* knock-in mice and in *Ikkα^−/−^* and *Ikkα^AA/AA^* BM chimeras [15], [28]. Similar effects were seen in *Baff^−/−^* mice [29] and revealed a crucial role for the non-canonical Baff-Baffr-Nik-Ikkα pathway in B-cell maturation and survival. Our observation that the Cd19^+^ B-cell population is also significantly decreased in the BM of *Ikkα^AA/AA^Apoe^−/−^*-transplanted *Apoe^−/−^* mice (Figure S1) suggests that the Ikkα kinase activity is also important in BM B-cell development. This corresponds to recent findings of Balkhi and colleagues, who identified a reduction in the Cd19^+^, B220^+^ and Cd19^+^B220^+^ B-cell population in the BM of kinase-dead *Ikkα* (*Ikkα^KA/KA^*) knock-in mice and *Ikkα^KA/KA^* BM chimeras, and also revealed less B220^+^Cd19^+^ B-cells in the BM of irradiated *Rag^−/−^* mice reconstituted with *Ikkα^−/−^* fetal liver cells [30]. Thus, our data support this important role for Ikkα kinase activity in early B-cell development in the BM, which was revealed to involve both canonical and non-canonical NF-κB pathways [30].

Secondly, we observed a consistent increase in the Cd62L^high^Cd44^low^ naive T-cell population in secondary lymphoid organs of *Ikkα^AA/AA^Apoe^−/−^* BM chimeras, whereas Cd62L^low^Cd44^high^ effector memory T-cells were decreased (Figure 1C). This corresponds with a previous observation of Mancino and colleagues, who revealed that Ikkα kinase activity in DCs is crucial for antigen-specific priming of naive T-cells in an *in vivo* delayed-type hypersensitivity model [31]. Similar effects on the ratio of naive *vs* effector memory T-cells were seen in mice deficient for *Nik*, the kinase activating Ikkα in the non-canonical NF-κB pathway [32]. Likewise, *Nik^aly/aly^* mice, which carry a natural Nik mutant (*Nik^aly^*) unable to bind Ikkα and associated with reduced NF-κB activation [33]–[35], presented with reduced T-cell effector cytokine expression, which was ascribed to a *Nik*-deficiency in thymic DCs rather than to an intrinsic T-cell defect [36]. Despite equal DC numbers in the thymus of *Nik^aly/aly^* and *Nik^+/+^* mice, thymic *Nik^aly/aly^* DCs showed a decreased expression of typical activation markers [36]. Similarly, also splenic *Nik^aly/aly^* DCs expressed a considerably lower level of MhcII compared to *Nik^aly/+^* DCs [37], suggesting an inability of *Nik^aly/aly^* DCs to deliver T-cell costimulatory signals and contribute to the development of effector T-cells [36]. In line with this, our *Ikkα^AA/AA^Apoe^−/−^* BM chimeras displayed a significantly reduced MhcII expression on splenic pDCs (Figure 1D). Very recently, an increased ratio of naive *vs* effector memory T-cells was also seen in *Nik^−/−^* BM chimeras, but was linked with a cell-intrinsic role of Nik in the generation or maintenance of effector memory T-cells [38]. Given these new findings together with the previous observations of Mancino *et al.* [31], the relative importance of cell-intrinsic *vs* DC-mediated effects on the increased ratio of naive *vs* effector memory T-cells in our *Ikkα^AA/AA^* BM chimeras remains to be investigated.

In addition to reduced effector memory T-cells, *Ikkα^AA/AA^(Apoe^−/−^)* BM chimeras displayed a significantly reduced T_reg_ population (Figure 1B, Figure S5). T_reg_ cells develop in the thymus and in peripheral sites from naive CD4^+^ T-cells. Initially, defective alternative NF-κB activation in the thymic stroma and a disorganized thymic structure in *Nik^aly/aly^* mice was associated with a defective establishment of self-tolerance and reduced T_reg_ numbers [39]. Later, a study of single *Nik^−/−^ vs* mixed Nik^−/−^/Nik^+/+^ BM chimeras indicated an additional cell-intrinsic role for Nik in the maintenance of peripheral T_reg_ T-cells [38]. Also, at least part of the T_reg_ defect in *Nik^aly/aly^* mice was attributed to the reduced capacity of *Nik^aly/aly^* DCs to trigger T_reg_ expansion and survival *in vitro* [37], and both NIK and IKKα were shown to be required in human DCs to trigger the development of T_reg_ cells from naive CD4^+^ T-cells *in vitro* [40]. Subsequently, pDC-triggered T_reg_ development from naive T-cells was associated with the induction of indoleamine 2,3-dioxygenase in pDCs by NIK-mediated non-canonical NF-κB activation [40]–[43]. With Ikkα a crucial player in this alternative NF-κB pathway, our data now directly confirm for the first time a role for haematopoietic Ikkα kinase activation in the generation of T_reg_ cells *in vivo*. However, the relative importance of T_reg_-intrinsic *vs* DC-mediated mechanisms remain to be investigated, both in the periphery as thymus, given that peripheral DCs can migrate into the thymus to actively contribute to thymic T_reg_ generation [44]. Of note, the reduced T_reg_ population in *Ikkα^AA/AA^Apoe^−/−^* BM chimeras could be associated with the increased serum levels of VLDL observed in these mice, as depletion of T_reg_ T-cells was recently discovered to enhance VLDL levels through reduced VLDL clearance [45].

Despite the clear effects of a BM-specific *Ikkα^AA/AA^Apoe^−/−^* knock-in on haematopoiesis and the enhanced VLDL levels, no differences were observed in the size, phenotype and cellular composition of atherosclerotic lesions in hyperlipidaemic *Apoe^−/−^* mice transplanted with *Ikkα^AA/AA^Apoe^−/−^* or *Ikkα^+/+^Apoe^−/−^* BM (Figure 2–5). The influence of the reduced B-cell population in the *Ikkα^AA/AA^Apoe^−/−^* BM chimeras on atherosclerosis is unclear. On the one hand, an atheroprogressive effect could be suggested based on the observation that a transplantation of *Ldlr^−/−^* mice with B-cell deficient (*μMT*) BM aggravated atherosclerosis [46] and also several other studies indicated an atheroprotective role for B-cells [1], [16]. However, different B-cell subsets have diverse functions in atherogenesis and B2 B-cells, in contrast to B1 B-cells, rather exacerbate atherosclerosis [16]. In this context, a lack of B2 B-cells, but a preserved B1 B-cell population was detected in mice with a deficiency of *Baff* [29] or *Baffr* [47], which can signal to NF-κB through Ikkα. This was associated with reduced atherosclerosis in *Baffr^−/−^Apoe^−/−^* or *Baffr^−/−^* BM-transplanted *Ldlr^−/−^* mice compared to controls [48], [49]. Although B-cell subsets were not examined in our study, Senftleben *et al.* predominantly found a reduction in the mature IgM^low^IgD^high^ B-cell population in *Ikkα^AA/AA^* mice and *Ikkα^AA/AA^* BM chimeras [15], with IgD being a surface marker of follicular B2 B-cells [16]. Thus, the reduced B-cell population in our *Ikkα^AA/AA^Apoe^−/−^* BM chimeras may by itself provide atheroprotective effects through a potential decrease in B2 B-cells. Regarding T-lymphocytes, effector memory T-cells are present in atherosclerotic lesions [1] and were shown to positively correlate with the extent of atherosclerosis in atherogenic mice, whereas naive T-cells were inversely correlated with plaque size [50]. Furthermore, T_reg_ T-cells have been shown to mediate atheroprotective functions [1], [26]. Therefore, pro-atherogenic effects of a reduction in the T_reg_ population in *Ikkα^AA/AA^Apoe^−/−^* BM chimeras may be compensated by atheroprotective effects of the observed relative increase of naive *versus* effector memory T-cells in these mice. Comparable results were seen upon deficiency of Cd40-Traf2/3/5 signalling in *Apoe^−/−^* mice, which did not affect atherosclerosis despite an increase in both atheroprogressive effector memory T-cells and atheroprotective T_reg_ cells in blood and secondary lymphoid organs [51]. Altogether, this suggests that simultaneous changes in lymphocyte subsets may completely balance individual atheroprogressive and -protective effects, without any net effect on atherosclerosis, similarly as observed in our study.

As macrophages play an important role in the uptake of modified lipids in atherosclerotic lesions, we examined the effect of an *Ikkα^AA/AA^* knock-in on macrophage intracellular lipid accumulation. However, no significant differences were observed *in vitro* or in atherosclerotic lesions *in vivo* (Figure 7), which is similar as previously observed for *Ldlr^−/−^* mice with a myeloid-specific deletion of *Ikkβ* [11].

Furthermore, the *Ikkα^AA/AA^* knock-in mutation was previously shown to reduce macrophage apoptosis upon bacterial infection, which was associated with prolonged LPS-triggered NF-κB p65 activation in BM-derived macrophages [19]. As apoptosis is an important process in atherogenesis, being atheroprotective in early stages of disease but associated with plaque necrosis and atheroprogression in later phases [52], we investigated lesional apoptosis in *Ikkα^AA/AA^Apoe^−/−^* and *Ikkα^+/+^Apoe^−/−^* BM chimeras. However, no significant differences were observed in cellular or macrophage apoptosis, and also necrotic core sizes were comparable (Figure 4). Furthermore, we did not observe a differential activity of NF-κB p65 upon LPS stimulation of *Ikkα^AA/AA^Apoe^−/−^ vs Ikkα^+/+^Apoe^−/−^* BM-derived macrophages *in vitro*, and even detected a reduced response in p65 activation upon atherogenic (i.e. Tnf-α, oxLDL) exposure (Figure 6A). Although these differential observations between our study and the one from Lawrence and colleagues [19] are unexpected at first sight, a major difference between the two studies is the use of *Apoe^−/−^* macrophages in our report. ApoE has been recognized as an important immunomodulator, which in macrophages promotes the anti-inflammatory M2 phenotype [53]. It is well-known that regulatory effects on NF-κB signalling behave cell type-specific, also in the context of Ikkα-mediated NF-κB regulation. For example, the *Ikkα^AA/AA^* knock-in mutation did not affect LPS-induced canonical NF-κB activation in BM-derived DCs [31], and even induced a small reduction in basal and LPS-induced NF-κB activation in B-cells [15]. Thus, the dissimilar effects of an *Ikkα^AA/AA^* knock-in mutation on LPS-induced NF-κB p65 activity in macrophages in our study compared to the one from Lawrence *et al.* [19] could be due to differential macrophage phenotypes induced by *Apoe*-deficiency or even by different culturing conditions. Furthermore, NF-κB regulatory mechanisms are often stimulus-dependent, as also exemplified by the observation that an *Ikkα^AA/AA^* knock-in did not affect Tnf-α-mediated NF-κB activation in fibroblasts or mammary epithelial cells [22]. This could additionally explain why in our study no prolonged p65 activity could be observed in Tnf-α- or oxLDL-stimulated *Ikkα^AA/AA^* macrophages *in vitro*.

Although the effect of Ikkα on NF-κB stability in macrophages was only described for the isoforms p65 and c-Rel [19], also p50 activity has been reported in atherosclerotic lesions [54]–[56]. Even more, it has been suggested that p50-p50 homodimers represent the main NF-κB activity during inflammation resolution, at least in a rat carrageenin-induced pleurisy model [8], which could correspond to the higher inflammatory phenotype of atherosclerotic lesions in *Ldlr^−/−^* mice with a haematopoietic *p50* deficiency [57]. Although beyond the scope of this study, it would be interesting to perform a detailed characterization of canonical NF-κB isoform activity in different stages of atherosclerosis and investigate a potential regulation by the IKKα kinase under these specific atherosclerotic conditions *in vivo*. Simultaneously, the activity of non-canonical NF-κB isoforms in the course of atherogenesis could be readdressed. Although a single study reported the absence of p52 and RelB activity in isolated human atherosclerotic plaque cells [55], a recent report identified a significant upregulation of IKKα and p52 during human monocyte-macrophage differentiation *in vitro*. This enables p52-mediated transcriptional repression under basal conditions, possibly preventing macrophage hyperactivation, but facilitates enhanced RelB-52 activity upon inflammation-induced RelB expression [58].

Our study did not reveal significant differences in inflammatory protein levels in the supernatants of Tnfα- or oxLDL-stimulated *Ikkα^AA/AA^Apoe^−/−^ vs Ikkα^+/+^Apoe^−/−^* BM-derived macrophages, with the exception of Il-12 which showed a significant increase in oxLDL-stimulated macrophages upon *Ikkα^AA/AA^* knock-in (Figure 6B, Figure S6). Furthermore, we could not detect significant differences in macrophage-related inflammatory cytokine and chemokine levels in the serum of atherosclerotic *Ikkα^AA/AA^Apoe^−/−^ vs Ikkα^+/+^Apoe^−/−^* BM chimeras (Figure 6C). Thus, despite the diverse roles of the Ikkα kinase in modulating gene expression in an NF-κB-dependent or -independent manner [4], [14], these functions do not seem strong enough in the context of atherogenesis to produce a major effect on systemic protein expression upon *Ikkα^AA/AA^* knock-in.

Furthermore, it is important to remember that the identification of novel IKKα substrates continuously extends the molecular pathways and biological processes affected by this kinase [4], [14]. Also, atherosclerosis is influenced by many leukocyte subsets [1], [2]. Therefore, it is conceivable that our overall zero effect of the BM-specific *Ikkα^AA^* mutation on atherosclerosis is at least partially the result of counterbalanced effects on different biological processes in macrophages or even different leukocyte subsets. For example, it would be interesting to study the effect of a DC- or T_reg_-specific *Ikkα^AA/AA^* mutation on atherogenesis. Also, both IKKα and IKKβ were shown to be important in neutrophil chemotaxis to HMGB1, a nuclear protein released by necrotic cells, but the functions of the IKKα kinase activity in neutrophil responses and molecular signalling in the context of inflammation and atherosclerosis have not yet been investigated. In addition, the role of IKKα in vascular cells remains to be investigated in more detail.

In conclusion, our data identify an important and previously unrecognized role for the haematopoietic Ikkα kinase activity in B- and T-cell homeostasis in conditions of atherosclerosis. However, the BM-specific *Ikkα^AA^* knock-in in atherosclerotic mice did not affect the size or phenotype of atherosclerotic lesions. This indicates that the diverse functions of Ikkα in haematopoietic cells may counterbalance each other or may not be strong enough to influence atherogenesis, and reveals that targeting haematopoietic *Ikkα* kinase activity alone may not represent a suitable therapeutic approach. Although the overall zero effect on atherosclerosis is surprising at first sight, it has been observed before that deficiency of proteins with an important role in inflammatory signalling and biological processes does not induce any changes in the size or composition of atherosclerotic lesions, as for example described for BM-deficiency of *Cd40 ligand* [59], [60] or *Traf6* [61]. Also, atherosclerosis was not affected in *Ldlr^−/−^* mice with a BM *p16^INK4a^*-deficiency [62], despite the fact that p16^INK4a^ is a regulator of macrophage activation and polarization and *p16^INK4a^*-deficiency reduces LPS-induced NF-κB activation in BM-derived macrophages [63]. Clearly, it would be interesting to address in the future the role of the IKKα kinase in atherosclerosis in different leukocyte subsets individually and in vascular cells.

## Supporting Information

Figure S1 **Effect of a bone marrow-specific** ***Ikkα^AA/AA^*** **knock-in on B- and T-cell populations in bone marrow and secondary lymphoid organs.** Shown is flow cytometric analysis of bone marrow, spleen and lymph nodes from *Apoe^−/−^* mice transplanted with *Ikkα^AA/AA^Apoe^−/−^* or *Ikkα^+/+^Apoe^−/−^* BM and receiving a high-cholesterol diet for 13 weeks. (A) Cd19^+^ B-cell population as percentage of Cd45^+^ leukocytes. (B–C) Cd19^+^ B-cell and Cd3^+^ T-cell populations as percentage of Cd45^+^ leukocytes, and Cd4^+^ and Cd8a^+^ T-cell subsets as percentage of Cd3^+^ T-cells in lymph nodes (B) and spleen (C). All graphs represent the mean ± SEM (n = 18–19), 2-tailed t-test, ***P<0.001.
(DOCX)

Figure S2 **Effect of a bone marrow-specific** ***Ikkα^AA/AA^*** **knock-in on T_reg_, naive and effector memory T-cells.** Shown are representative dot plots of the FACS-based gating strategy of T-cell subpopulations within splenic leukocytes from *Ikkα^+/+^Apoe^−/−^* and *Ikkα^AA/AA^Apoe^−/−^* BM chimeras after 13 weeks of high-cholesterol diet. (**A**) Within the Cd3^+^ T-cell population, T_reg_ cells were defined as Cd4^+^Cd25^+^Foxp3^+^ cells. (**B**) Within the Cd3^+^ T-cell population, naive T-cells were defined as Cd44^low^Cd62L^high^ T-cells and effector memory T-cells as Cd44^high^Cd62L^low^ T-cells. (A,B) Percentages indicate the % from the Cd3^+^ T-cell population.
(DOCX)

Figure S3 **Effect of a bone marrow-specific** ***Ikkα^AA/AA^*** **knock-in on central memory T-cells.** Shown is flow cytometric analysis of Cd44^high^Cd62L^high^ central memory T-cells in spleen and lymph nodes from *Apoe^−/−^* mice transplanted with *Ikkα^AA/AA^Apoe^−/−^* or *Ikkα^+/+^Apoe^−/−^* BM and receiving a high-cholesterol diet for 13 weeks. Data are represented as percentage of Cd3^+^ T-cells (left) and as percentage of Cd45^+^ leukocytes (right). Graphs represent the mean ± SEM (n = 18–19), 2-tailed t-test, *P<0.05, **P<0.01, ***P<0.001.
(DOCX)

Figure S4 **Effect of a bone marrow-specific** ***Ikkα^AA/AA^*** **knock-in on B- and T-cell populations in a non-atherosclerotic context.** Shown is flow cytometric analysis of spleen and lymph nodes from C57BL/6 mice transplanted with *Ikkα^AA/AA^* or *Ikkα^+/+^* BM. Dead cells were excluded using Sytox Blue. (**A**) B220^+^ B-cell population as percentage of leukocytes, and the total number of B-cells in spleen and lymph nodes. (**B**) Cd4^+^ and Cd8a^+^ T-cell subsets as percentage of leukocytes, and as percentage of Cd3^+^ T-cells. (**C**) Total number of Cd3^+^Cd4^+^ and Cd3^+^Cd8a^+^ T-cell subsets, and total leukocyte number in spleen and lymph nodes. All graphs represent the mean ± SEM (n = 5); 2-tailed t-test; *P<0.05, **P<0.01, ***P<0.001.
(DOCX)

Figure S5 **Effect of a bone marrow-specific** ***Ikkα^AA/AA^*** **knock-in on T_reg_-cells in a non-atherosclerotic context.** Shown is flow cytometric analysis of T_reg_-cells in lymph nodes from C57BL/6 mice transplanted with *Ikkα^AA/AA^* or *Ikkα^+/+^* BM. Dead cells were excluded using Sytox Blue. (**A**) Cd4^+^Foxp3^+^ T_reg_-cells as percentage of leukocytes (left), and Cd4^+^Cd25^+^Foxp3^+^ T_reg_-cells as percentage of Cd4^+^ T-cells (right). (**B**) Total numbers of Cd4^+^Foxp3^+^ T_reg_-cells (left), and total numbers of Cd4^+^Cd25^+^Foxp3^+^ T_reg_-cells. Graphs represent the mean ± SEM (n = 5), 2-tailed t-test, *P<0.05, **P<0.01, ***P<0.001.
(DOCX)

Figure S6 **Effect of** ***Ikkα^AA/AA^*** **knock-in on cytokine secretion from BM-derived macrophages.** Shown are cytokine concentrations of Il-10 and Il-12p70 in the supernatants of *Ikkα^AA/AA^Apoe^−/−^* or *Ikkα^+/+^Apoe^−/−^* BM-derived macrophages, unstimulated or after stimulation for 24 h with 10 ng/ml Tnf-α or 50 µg/ml oxLDL, as indicated. Graphs represent mean ± SEM (n = 9 from 3 independent experiments); 2-way ANOVA with Bonferroni post-test, *P<0.05, **P<0.01.
(DOCX)
