# Supplementary material for: Bone Marrow-Specific Knock-In of a Non-Activatable Ikkα Kinase Mutant Influences Haematopoiesis but Not Atherosclerosis in Apoe-Deficient Mice
Source: PLoS One. 2014 Feb 3;9(2):e87452. doi: 10.1371/journal.pone.0087452 (PMC3911989; doi:10.1371/journal.pone.0087452)
Supplement: Figure S1 — Effect of a bone marrow-specific IkkαAA/AA knock-in on B- and T-cell populations in bone marrow and secondary lymphoid organs. Shown is flow cytometric analysis of bone marrow, spleen and lymph nodes from Apoe−/− mice transplanted with IkkαAA/AAApoe−/− or Ikkα+/+Apoe−/− BM and receiving a high-cholesterol diet for 13 weeks. (A) Cd19+ B-cell population as percentage of Cd45+ leukocytes. (B–C) Cd19+ B-cell and Cd3+ T-cell populations as percentage of Cd45+ leukocytes, and Cd4+ and Cd8a+ T-cell subsets as percentage of Cd3+ T-cells in lymph nodes (B) and spleen (C). All graphs represent the mean ± SEM (n = 18–19), 2-tailed t-test, ***P<0.001. (DOCX) [file pone.0087452.s001.docx]

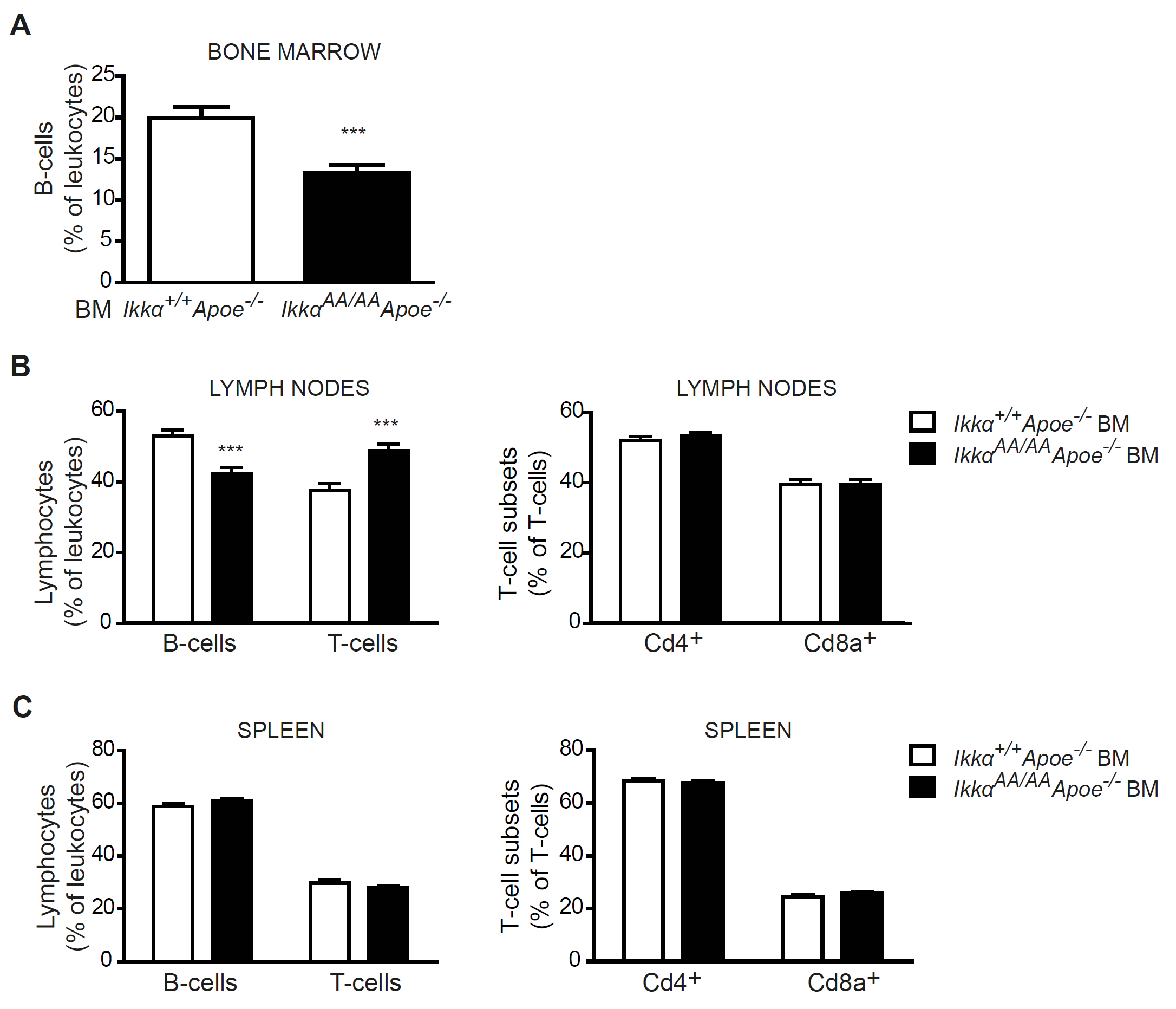


**Figure S1. Effect of a bone marrow-specific *Ikkα^AA/AA^* knock-in on B- and T-cell populations in bone marrow and secondary lymphoid organs.** Shown is flow cytometric analysis of bone marrow, spleen and lymph nodes from *Apoe^-/-^*  mice transplanted with *Ikkα^AA/AA^Apoe^-/-^*  or *Ikkα^+/+^Apoe^-/-^*  BM and receiving a high-cholesterol diet for 13 weeks. (**A**) Cd19^+^ B-cell population as percentage of Cd45^+^ leukocytes. **(B-C)** Cd19^+^ B-cell and Cd3^+^ T-cell populations as percentage of Cd45^+^ leukocytes, and Cd4^+^ and Cd8a^+^ T-cell subsets as percentage of Cd3^+^ T-cells in lymph nodes (B) and spleen (C). All graphs represent the mean ± SEM (n=18-19), 2-tailed t-test, ***P<0.001.
