# Supplementary material for: Bone Marrow-Specific Knock-In of a Non-Activatable Ikkα Kinase Mutant Influences Haematopoiesis but Not Atherosclerosis in Apoe-Deficient Mice
Source: PLoS One. 2014 Feb 3;9(2):e87452. doi: 10.1371/journal.pone.0087452 (PMC3911989; doi:10.1371/journal.pone.0087452)
Supplement: Figure S2 — Effect of a bone marrow-specific IkkαAA/AA knock-in on Treg, naive and effector memory T-cells. Shown are representative dot plots of the FACS-based gating strategy of T-cell subpopulations within splenic leukocytes from Ikkα+/+Apoe−/− and IkkαAA/AAApoe−/− BM chimeras after 13 weeks of high-cholesterol diet. (A) Within the Cd3+ T-cell population, Treg cells were defined as Cd4+Cd25+Foxp3+ cells. (B) Within the Cd3+ T-cell population, naive T-cells were defined as Cd44lowCd62Lhigh T-cells and effector memory T-cells as Cd44highCd62Llow T-cells. (A,B) Percentages indicate the % from the Cd3+ T-cell population. (DOCX) [file pone.0087452.s002.docx]

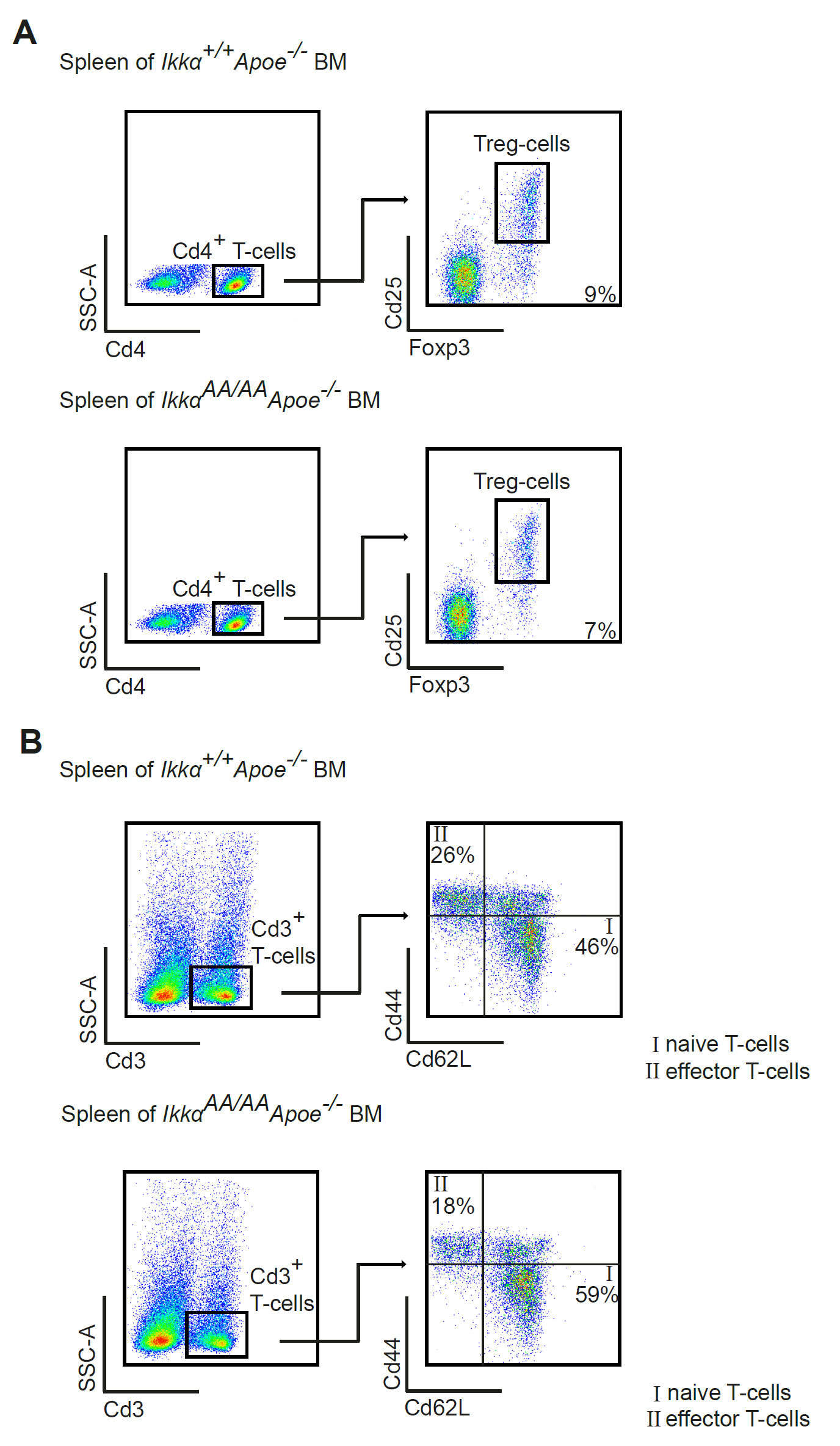


**Figure S2. Effect of a bone marrow-specific *Ikkα^AA/AA^* knock-in on T_reg_, naive and effector memory T-cells.** Shown are representative dot plots of the FACS-based gating strategy of T-cell subpopulations within splenic leukocytes from *Ikkα^+/+^Apoe^-/-^* and *Ikkα^AA/AA^Apoe^-/-^* BM chimeras after 13 weeks of high-cholesterol diet. **(A)** Within the Cd3^+^ T-cell population, T_reg_ cells were defined as Cd4^+^Cd25^+^Foxp3^+^ cells. **(B)** Within the Cd3^+^ T-cell population, naive T-cells were defined as Cd44^low^Cd62L^high^ T-cells and effector memory T-cells as Cd44^high^Cd62L^low^ T-cells. (A,B) Percentages indicate the % from the Cd3^+^ T-cell population.
