# Supplementary material for: Bone Marrow-Specific Knock-In of a Non-Activatable Ikkα Kinase Mutant Influences Haematopoiesis but Not Atherosclerosis in Apoe-Deficient Mice
Source: PLoS One. 2014 Feb 3;9(2):e87452. doi: 10.1371/journal.pone.0087452 (PMC3911989; doi:10.1371/journal.pone.0087452)
Supplement: Figure S3 — Effect of a bone marrow-specific IkkαAA/AA knock-in on central memory T-cells. Shown is flow cytometric analysis of Cd44highCd62Lhigh central memory T-cells in spleen and lymph nodes from Apoe−/− mice transplanted with IkkαAA/AAApoe−/− or Ikkα+/+Apoe−/− BM and receiving a high-cholesterol diet for 13 weeks. Data are represented as percentage of Cd3+ T-cells (left) and as percentage of Cd45+ leukocytes (right). Graphs represent the mean ± SEM (n = 18–19), 2-tailed t-test, *P<0.05, **P<0.01, ***P<0.001. (DOCX) [file pone.0087452.s003.docx]

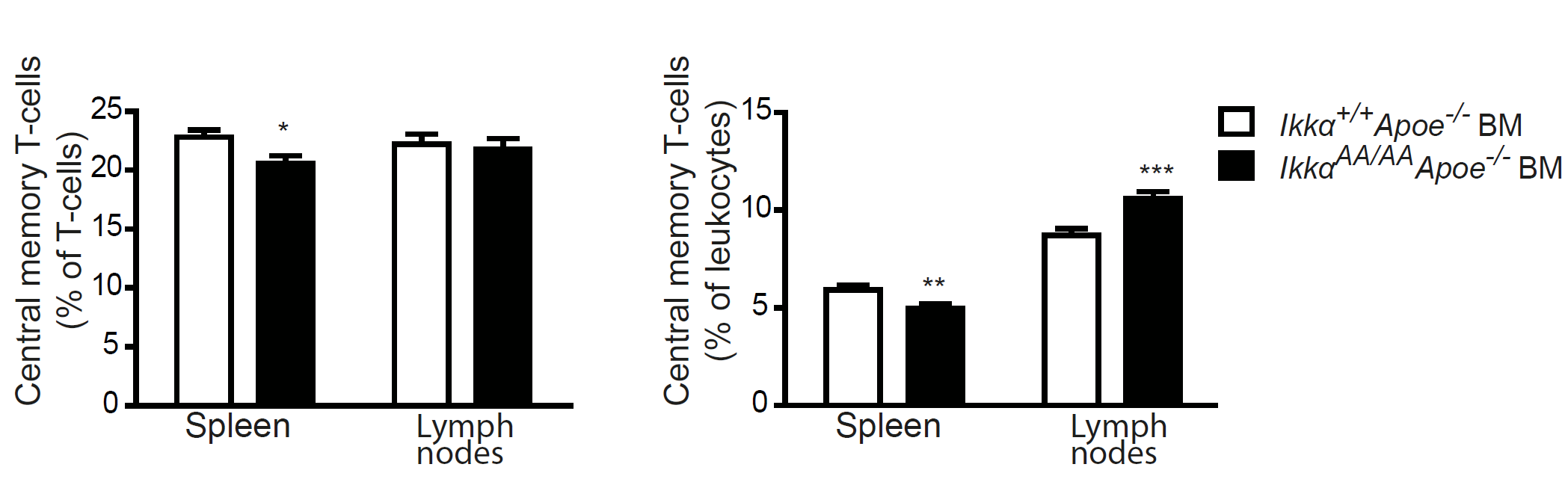


**Figure S3.** **Effect of a bone marrow-specific *Ikkα^AA/AA^* knock-in on central memory T-cells.** Shown is flow cytometric analysis of Cd44^high^Cd62L^high^ central memory T-cells in spleen and lymph nodes from *Apoe^-/-^*  mice transplanted with *Ikkα^AA/AA^Apoe^-/-^*  or *Ikkα^+/+^Apoe^-/-^*  BM and receiving a high-cholesterol diet for 13 weeks. Data are represented as percentage of Cd3^+^ T-cells (left) and as percentage of Cd45^+^ leukocytes (right). Graphs represent the mean ± SEM (n=18-19), 2-tailed t-test, *P<0.05, **P<0.01, ***P<0.001.
