# Supplementary material for: Bone Marrow-Specific Knock-In of a Non-Activatable Ikkα Kinase Mutant Influences Haematopoiesis but Not Atherosclerosis in Apoe-Deficient Mice
Source: PLoS One. 2014 Feb 3;9(2):e87452. doi: 10.1371/journal.pone.0087452 (PMC3911989; doi:10.1371/journal.pone.0087452)
Supplement: Figure S4 — Effect of a bone marrow-specific IkkαAA/AA knock-in on B- and T-cell populations in a non-atherosclerotic context. Shown is flow cytometric analysis of spleen and lymph nodes from C57BL/6 mice transplanted with IkkαAA/AA or Ikkα+/+ BM. Dead cells were excluded using Sytox Blue. (A) B220+ B-cell population as percentage of leukocytes, and the total number of B-cells in spleen and lymph nodes. (B) Cd4+ and Cd8a+ T-cell subsets as percentage of leukocytes, and as percentage of Cd3+ T-cells. (C) Total number of Cd3+Cd4+ and Cd3+Cd8a+ T-cell subsets, and total leukocyte number in spleen and lymph nodes. All graphs represent the mean ± SEM (n = 5); 2-tailed t-test; *P<0.05, **P<0.01, ***P<0.001. (DOCX) [file pone.0087452.s004.docx]

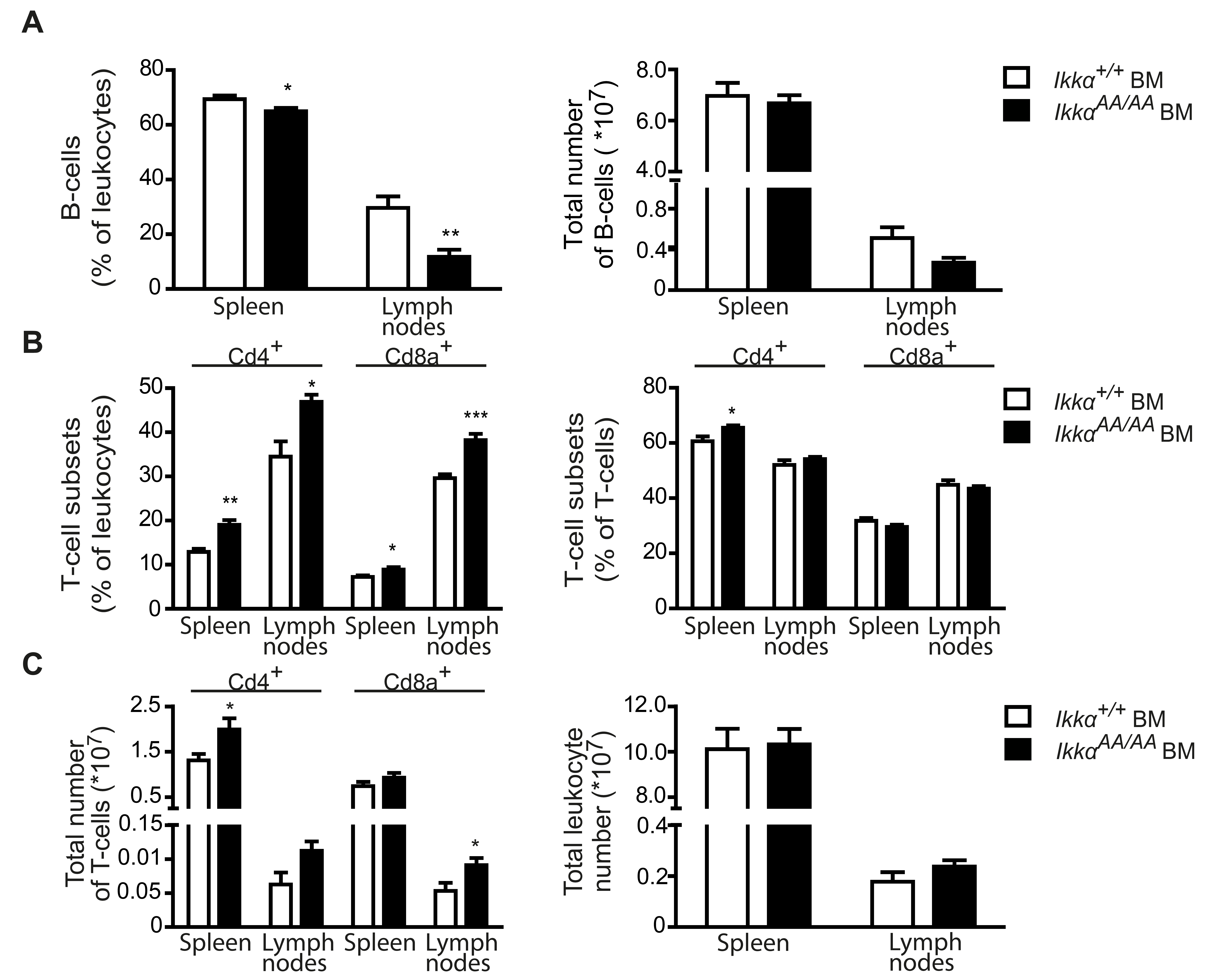


**Figure S4.** **Effect of a bone marrow-specific *Ikkα^AA/AA^* knock-in on B- and T-cell populations in a non-atherosclerotic context.** Shown is flow cytometric analysis of spleen and lymph nodes from C57BL/6 mice transplanted with *Ikkα^AA/AA^* or *Ikkα^+/+^* BM. Dead cells were excluded using Sytox Blue. **(A)** B220^+^ B-cell population as percentage of leukocytes, and the total number of B-cells in spleen and lymph nodes. **(B)** Cd4^+^ and Cd8a^+^ T-cell subsets as percentage of leukocytes, and as percentage of Cd3^+^ T-cells. **(C)** Total number of Cd3^+^Cd4^+^ and Cd3^+^Cd8a^+^ T-cell subsets, and total leukocyte number in spleen and lymph nodes. All graphs represent the mean ± SEM (n=5); 2-tailed t-test; *P<0.05, **P<0.01, ***P<0.001.
