# Supplementary material for: Bone Marrow-Specific Knock-In of a Non-Activatable Ikkα Kinase Mutant Influences Haematopoiesis but Not Atherosclerosis in Apoe-Deficient Mice
Source: PLoS One. 2014 Feb 3;9(2):e87452. doi: 10.1371/journal.pone.0087452 (PMC3911989; doi:10.1371/journal.pone.0087452)
Supplement: Figure S5 — Effect of a bone marrow-specific IkkαAA/AA knock-in on Treg-cells in a non-atherosclerotic context. Shown is flow cytometric analysis of Treg-cells in lymph nodes from C57BL/6 mice transplanted with IkkαAA/AA or Ikkα+/+ BM. Dead cells were excluded using Sytox Blue. (A) Cd4+Foxp3+ Treg-cells as percentage of leukocytes (left), and Cd4+Cd25+Foxp3+ Treg-cells as percentage of Cd4+ T-cells (right). (B) Total numbers of Cd4+Foxp3+ Treg-cells (left), and total numbers of Cd4+Cd25+Foxp3+ Treg-cells. Graphs represent the mean ± SEM (n = 5), 2-tailed t-test, *P<0.05, **P<0.01, ***P<0.001. (DOCX) [file pone.0087452.s005.docx]

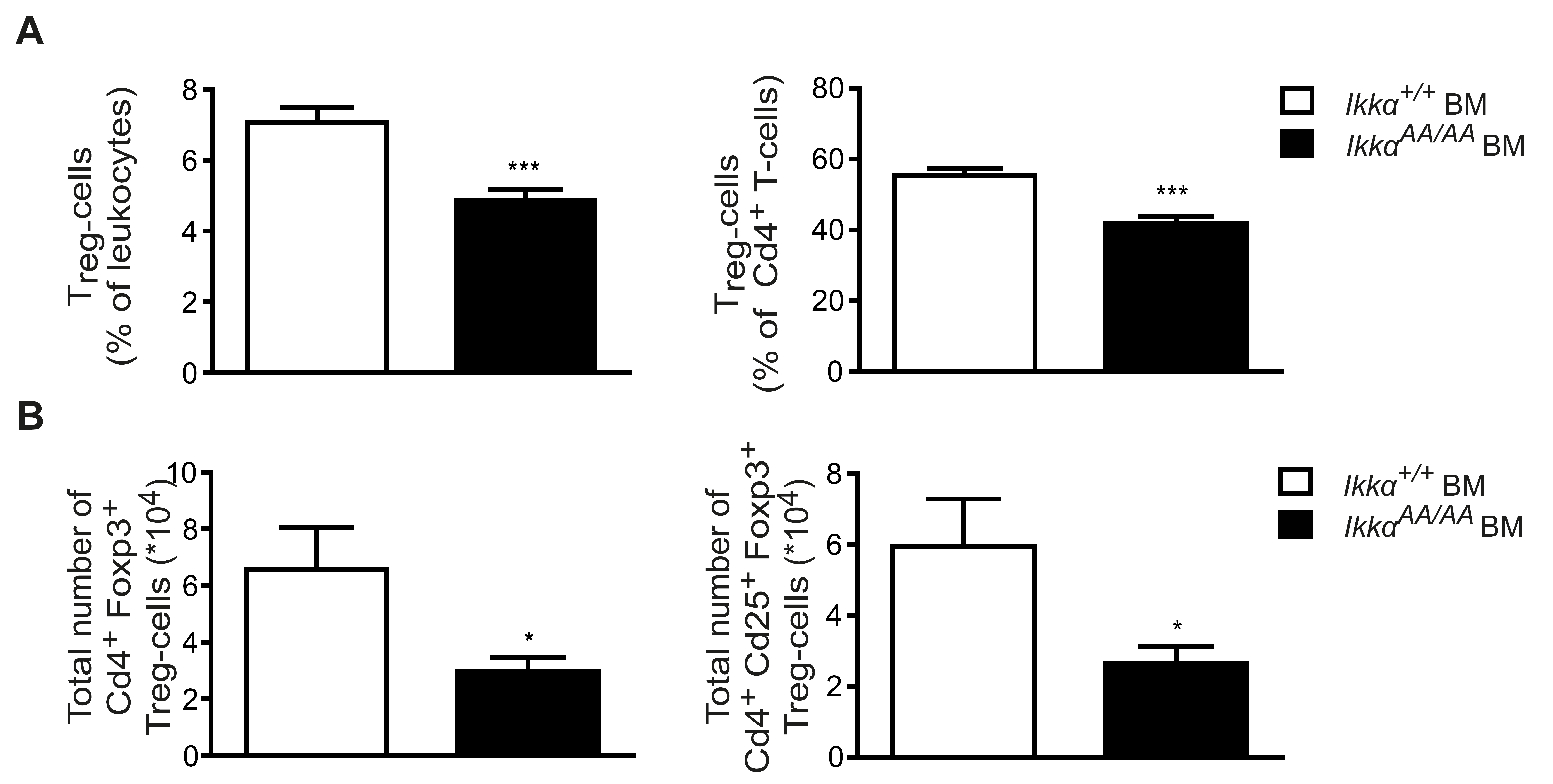


**Figure S5.** **Effect of a bone marrow-specific *Ikkα^AA/AA^* knock-in on T_reg_-cells in a non-atherosclerotic context.** Shown is flow cytometric analysis of T_reg_-cells in lymph nodes from C57BL/6 mice transplanted with *Ikkα^AA/AA^* or *Ikkα^+/+^*  BM. Dead cells were excluded using Sytox Blue. **(A)** Cd4^+^Foxp3^+^ T_reg_-cells as percentage of leukocytes (left), and Cd4^+^Cd25^+^Foxp3^+^ T_reg_-cells as percentage of Cd4^+^ T-cells (right). **(B)** Total numbers of Cd4^+^Foxp3^+^ T_reg_-cells (left), and total numbers of Cd4^+^Cd25^+^Foxp3^+^ T_reg_-cells. Graphs represent the mean ± SEM (n=5), 2-tailed t-test, *P<0.05, **P<0.01, ***P<0.001.
