# Supplementary material for: Bone Marrow-Specific Knock-In of a Non-Activatable Ikkα Kinase Mutant Influences Haematopoiesis but Not Atherosclerosis in Apoe-Deficient Mice
Source: PLoS One. 2014 Feb 3;9(2):e87452. doi: 10.1371/journal.pone.0087452 (PMC3911989; doi:10.1371/journal.pone.0087452)
Supplement: Figure S6 — Effect of IkkαAA/AA knock-in on cytokine secretion from BM-derived macrophages. Shown are cytokine concentrations of Il-10 and Il-12p70 in the supernatants of IkkαAA/AAApoe−/− or Ikkα+/+Apoe−/− BM-derived macrophages, unstimulated or after stimulation for 24 h with 10 ng/ml Tnf-α or 50 µg/ml oxLDL, as indicated. Graphs represent mean ± SEM (n = 9 from 3 independent experiments); 2-way ANOVA with Bonferroni post-test, *P<0.05, **P<0.01. (DOCX) [file pone.0087452.s006.docx]

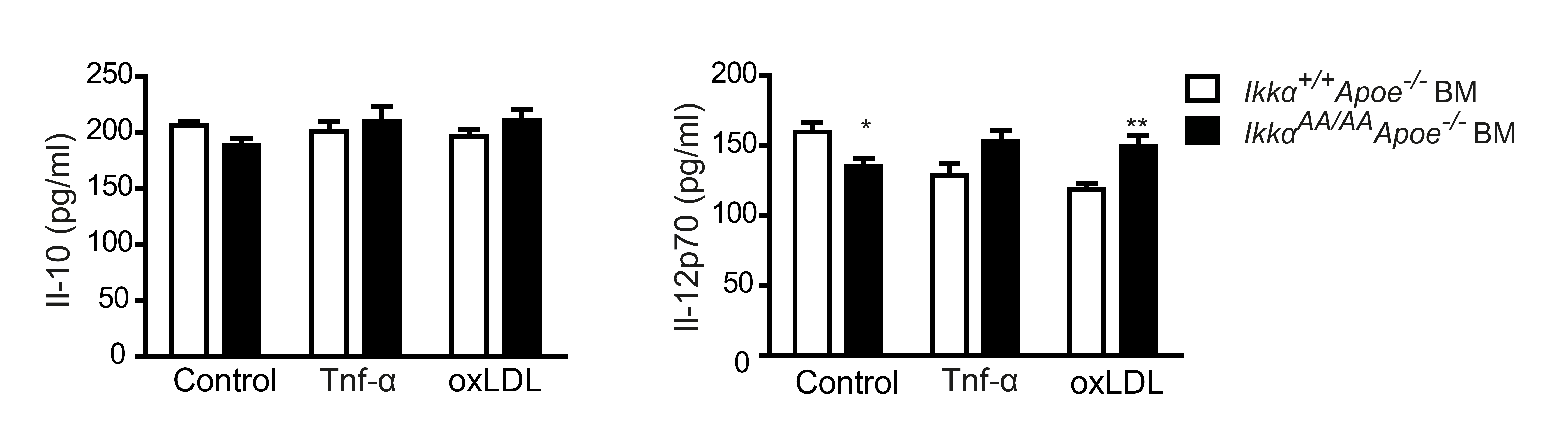


**Figure S6. Effect of *Ikkα^AA/AA^* knock-in on cytokine secretion from BM-derived macrophages.** Shown are cytokine concentrations of Il-10 and Il-12p70 in the supernatants of *Ikkα^AA/AA^Apoe^-/-^*  or *Ikkα^+/+^Apoe^-/-^*  BM-derived macrophages, unstimulated or after stimulation for 24 h with 10 ng/ml Tnf-α or 50 µg/ml oxLDL, as indicated. Graphs represent mean ± SEM (n=9 from 3 independent experiments); 2-way ANOVA with Bonferroni post-test, *P<0.05, **P<0.01.
